# Supplementary material for: Reductive inactivation of the hemiaminal pharmacophore for resistance against tetrahydroisoquinoline antibiotics
Source: Nat Commun. 2021 Dec 6;12:7085. doi: 10.1038/s41467-021-27404-3 (PMC8648761; doi:10.1038/s41467-021-27404-3)
Supplement: Supplementary file 1 — Supplementary Information [file 41467_2021_27404_MOESM1_ESM.pdf]

Supplementary Information

Reductive inactivation of the hemiaminal pharmacophore for resistance against tetrahydroisoquinoline antibiotics

Wan-Hong Wen[1], Yue Zhang[1], Ying-Ying Zhang[1], Qian Yu[2], Chu-Chu Jiang[2], Man-Cheng Tang[1], Jin-Yue Pu[1], Lian Wu[1], Yi-Lei Zhao[2], Ting Shi[2]*, Jiahai Zhou[3]*, and Gong-Li Tang[1,4]*

[1]State Key Laboratory of Bio-organic and Natural Products Chemistry, Center for Excellence in Molecular Synthesis, Shanghai Institute of Organic Chemistry, University of Chinese Academy of Sciences, Chinese Academy of Sciences, 345 Lingling Road, Shanghai 200032, China

[2]State Key Laboratory of Microbial Metabolism, Joint International Research Laboratory of Metabolic and Developmental Sciences, School of Life Sciences and Biotechnology, Shanghai Jiao Tong University, Shanghai 200240, China.

[3]CAS Key Laboratory of Quantitative Engineering Biology, Shenzhen Institute of Synthetic Biology, Shenzhen Institute of Advanced Technology, Chinese Academy of Sciences, Shenzhen 518055, China.

[4]School of Chemistry and Material Sciences, Hangzhou Institute for Advanced Study, University of Chinese Academy of Sciences, 1 Sub-lane Xiangshan, Hangzhou 310024, China

*Correspondence: Gong-Li Tang, Email: gltang@sioc.ac.cn

Jiahai Zhou, Email: jiahai@siat.ac.cn

Ting Shi, Email: tshi@sjtu.edu.cn

Contents

Supplementary Tables 4

Supplementary Table 1. Strains and plasmids used in this study. 4

Supplementary Table 2. List of PCR primers used in this study. 6

Supplementary Table 3. Statistics of X-ray crystallographic data collection and model refinements. 7

Supplementary Table 4. NMR spectroscopic data for **18.** 8

Supplementary Table 5. NMR spectroscopic data for **19.** 9

Supplementary Table 6. Information of homologous proteins selected by blastP in this study. 10

Supplementary Figures 11

Supplementary Figure 1. Construction and verification of *napW* gene-knockout mutant (*ΔnapW*, TG3022) of *S. lusitanus* NRRL 8034 11

Supplementary Figure 2. Construction and verification of *homW* (*ΔhomW*, TG3023) and dual-gene (*ΔnapW&ΔhomW,* TG3024) gene-knockout mutants of *S. lusitanus* NRRL 8034 11

Supplementary Figure 3. Overproduction in *E. coli* and purification of two enzymes utilized in this study. 12

Supplementary Figure 4. Cartoon representation of chain A of NapW structure 12

Supplementary Figure 5. Overlapping for structures of NapW-NADPH complex and NapW. 13

Supplementary Figure 6. Overproduction in *E. coli* and purification of NapW and mutants utilized in this study 13

Supplementary Figure 7. Catalytic activity of the NapW mutants to NDM detected by HPLC.. 14

Supplementary Figure 8. The potential energy surface obtained from umbrella sampling.. 14

Supplementary Figure 9. The RMSD values of MD simulations in WT and D165A mutant systems. 15

Supplementary Figure 10. The RMSF values of MD simulations in WT and D165A mutant.. 15

Supplementary Figure 11. The probability of water number within 3.4 Å of O3 atom of NDM in MD simulations. 16

Supplementary Figure 12. Analysis of NapW/BmGDH cascade reaction. 17
Supplementary Figure 13. NOEs (indicated by red arrows) determined the stereochemistry of compound **2**. 18
Supplementary Figure 14. Overproduction in *E. coli* and purification of SfmO1, homO1a and homO1b utilized in this study...19
Supplementary Figure 15. HR-MS/MS analysis of **16** 20
Supplementary Figure 16. HR-MS/MS analysis of **17**. 21
Supplementary Figure 17. HR-MS/MS analysis of ET-745 (**8**)........................22
Supplementary Figure 18. Structure characterization data of **18**.. 26
Supplementary Figure 19. Structure characterization data of **19**.......................30
Supplementary Figure 20. Fermentation analysis of LMM producing strain, *S. candidus* LL-AP191.. 31
Supplementary Figure 21. Sequence similarity network (SSN) analysis of NapW-homologues. 31
Supplementary Figure 22. Overproduction in *E. coli* and purification of SDR utilized in this study 32
Supplementary Figure 23. Enzymatic assays of NapW and homologous proteins towards **1** were detected by HPLC analysis...33
Supplementary Figure 24. Enzymatic assays of NapW and homologous proteins towards **16** were detected by HPLC-MS. 34
Supplementary Figure 25. Enzymatic assays of NapW and homologous proteins towards **7** were detected by HPLC analysis...35
Supplementary Figure 26. Enzymatic assays of NapW and homologous proteins towards **18** were detected by HPLC analysis...36
Supplementary References...37

Supplementary Tables

Supplementary Table 1. Strains and plasmids used in this study.

**Strains**	**Characteristics**	**References**
E. coli DH5α	Host for general cloning	Invitrogen
E. coli BL21 (DE3)	Host for protein expression	Invitrogen
E. coli Rosetta (DE3)	Host for protein expression	Novagen
E. coli S17-1	Donor strain for conjugation between *E. coli* and *S. lusitanus* NRRL 8034	4
S. lusitanus NRRL 8034	Wild type, NDM producing strain	NRRL
S. lavendulae NRRL 11002	Wild type, SFM producing strain	NRRL
S. candidus LL-AP191 (NRRL 3110)	Wild type, LMM producing strain	NRRL
S. lusitanus TG3022	Δ *napW* gene knockout mutant	This work
S. lusitanus TG3023	Δ *homW* gene knockout mutant	This work
S. lusitanus TG3024	Δ *napW& homW* gene knockout mutant	This work
**Plasmids**		
pMD19-T	Ap^R, *E. coli* subcloning vector	Takara
pKC1139	Am^R, *E. coli-Streptomyces* shuttle vector for gene inactivation	4
pET28a	Km^R, Protein expression in *E. coli*	Invitrogen
pET37b	Km^R, Protein expression in *E. coli*	Invitrogen
pRSF-BmGDH	Expression vector for D-Glucose dehydrogenase from Bacillus megaterium (BmGDH)	5
pTG3032	pKC1139 derivative for gene inactivation of *napW*	This work
pTG3046	pKC1139 derivative for gene inactivation of *homW*	This work
pTG3044	pET37b derivative containing gene *napW* for protein expression in *E. coli*	This work
pTG3045	pET28a derivative containing gene *napW* for protein expression in *E. coli*	This work
pTG3047	pET28a derivative containing gene *sfmO1* for protein expression in *E. coli*	This work
pTG3048	pET37b derivative containing gene *homO1a* for protein expression in *E. coli*	This work

pTG3049	pET37b derivative containing gene *homO1b* for protein expression in *E. coli*	This work
pTG3050	pET37b derivative containing gene *slvW1* for protein expression in *E. coli*	This work
pTG3051	pET37b derivative containing gene *slvW2* for protein expression in *E. coli*	This work
pTG3052	pET37b derivative containing gene SDR-Pb for protein expression in *E. coli*	This work
pTG3053	pET37b derivative containing gene SDR-Cc for protein expression in *E. coli*	This work
pTG3054	pET37b derivative containing gene SDR-Rm for protein expression in *E. coli*	This work
pTG3055	pET37b derivative containing gene SDR-Mb for protein expression in *E. coli*	This work
pTG3056	pET37b derivative containing gene SDR-Li for protein expression in *E. coli*	This work
pTG3057	pET37b derivative containing gene SDR-Cs for protein expression in *E. coli*	This work
pTG3058	pET37b derivative containing gene SDR-Ss for protein expression in *E. coli*	This work
pTG3059	pET37b derivative containing mutated *napW* (E113A) gene for protein expression	This work
pTG3060	pET37b derivative containing mutated *napW* (W213A) gene for protein expression	This work
pTG3061	pET37b derivative containing mutated *napW* (R176A) gene for protein expression	This work
pTG3062	pET37b derivative containing mutated *napW* (D165A) gene for protein expression	This work
pTG3063	pET37b derivative containing mutated *napW* (N177A) gene for protein expression	This work

Abbreviations: Ap^R, ampicillin resistance; Am^R, apramycin resistance; Km^R, kanamycin resistance.

Supplementary Table 2. List of PCR primers used in this study.

Primers	Sequences	Application
napW-L-for	GAATTCTCTGATGGCCGGTGTCTCCAAG	Gene knockout
napW-L-rev	TCTAGACAGCTCGCTCAGGAGTACGG	
napW-R-for	TCTAGACGTCTCGGACCGGTTGTACTC	
napW-R-rev	AAGCTTCCGAGTACGACGAGGAACACGTG	
napW-gt-for	GAACAGCGCGGTCACCGATACC	Genotype validation
napW-gt-rev	CGACTGGACGAGTACTGCACACCGT	
homW-L-for	GAATTCTACTCCTCGCGCTGTTCGACGCTG	Gene knockout
homW-L-rev	TCTAGAGACGTCACCGGCTACCGCTGAC	
homW-R-for	TCTAGACGGCTGTGTCATTCACGGCCTCC	
homW-R-rev	AAGCTTCGACGAAGGAGTCAGCAGCATGG	
homW-gt-for	CAGGACGTCCAGCGAGCCGAAGG	Genotype validation
homW-gt-rev	GAAGCGCCCGCGGTCACTGAGG	
homW-for	CATATGACACAGCCGTTGCGGGACAAGG	Protein expression
homW-rev	AAGCTTGCGGTAGCCGGTGACGTCGGCCG GGCGGCCCGCGTCCTGC	
SlvW1-for	CATATGACAGGGTCATCGAAAGGTCCGC	Protein expression
SlvW1-rev	AAGCTTGCGATACCCCGTGACGTCG	
SlvW2-for	CATATGGATGACATGAGCAACGAGGACA	Protein expression
SlvW2-rev	AAGCTTGCGGTAGTCGTCAGGGGAGGC	
NapW-E113A-for	GGGAGGTGCCCGACTGTTCGAGTTCGACA A	Site-directed mutation
NapW-E113A-rev	ACAGTCGGGCACCTCCCCAGACGTCGTTGA	
NapW-D165A-for	GATGACCGCGGGGACGGCCGCGTACAACG G	Site-directed mutation
NapW-D165A-rev	CCGTCCCCGCGGTCATCTCCACCACGAGTC	
NapW-R176A-for	CCACTACGCGAACTCGTACTTCTACGACCT	Site-directed mutation
NapW- R176A -rev	ACGAGTTCGCGTAGTGGCTGCCGTTGTACG	
NapW- N177A-for	CTACCGCGCGTCGTACTTCTACGACCTGGT	Site-directed mutation
NapW- N177A-rev	AGTACGACGCGCGGTAGTGGCTGCCGTTGT	
NapW-W213A-for	CGGTGACGCTCACGCCGGGCGCGATGCGTT CGGAGATGATGCT	Site-directed mutation
NapW-W213A -rev	AGCATCATCTCCGAACGCATCGCGCCCGGC GTGAGCGTCACCG	
HomO1a-for	TTTAAGAAGGAGATATACATATGGTGACGG GAACTGTCACCTTTG	Site-directed mutation
HomO1a-rev	TCGAGTGCGGCCGCAAGCTTGCGGTAGTCG TCCGGCGAGGCGTC	
HomO1b-for	TTTAAGAAGGAGATATACATATGACGTGGTC GCCCGATCC	Site-directed mutation
HomO1b-rev	TCGAGTGCGGCCGCAAGCTTGCGGTCGTGC CAGCCGTCCGGCCGGGTTCC	
SfmO1-for	ATATGAATTCCATATGACCGACGGCGTCCGC AC	Protein expression
HomO1-rev	ATATAAGCTTTTACTCGAGCAAGGGGGTAC CCAGCGGTC-	

Supplementary Table 3. Statistics of X-ray crystallographic data collection and model refinements.

	**NapW**	**NapW-NADPH complex**
**Data collection**		
Wavelength (Å)	0.97853	0.97918
Space group	*P*2	$P2_1$
Cell dimension		
a，b，c (Å)	117.149，134.438，117.216	116.42，116.04，134.76
α，β，γ (°)	90.000，90.041，90.000	90.00，90.04，90.00
Resolution (Å)	50.00-2.08 (2.12-2.08)	58.27-2.00 (2.05-2.00)
R_{merge} (%)	14.2 (99.7)	8.2 (84.6)
R_{meas}(%)	15.4 (107.7)	8.9 (92.0)
I/σ(I)	10.778 (2.200)	12.9 (2.4)
Completeness (%)	100.0 (100.0)	99.0 (99.4)
Redundancy	6.9 (7.0)	6.8（6.6）
Unique reflections	216044	238915
$CC_{1/2}$	0.990 (0.725)	0.999 (0.836)
**Structure refinement**		
Resolution (Å)	41.332 - 2.083	58.269 - 2.000
No. reflections	213192	238769
R_{work}/R_{free} (%)	21.02/24.91	17.75/20.17
No. atoms	18962	21109
Protein	17583	18693
Ligand		768
Water	1379	1648
Root mean square deviations		
bonds (Å)	0.0092	0.007
angles (Å)	0.93	1.16
B factor, overall (Å^2)	22.2	35.8
B factor, protein atoms (Å^2)	21.8	35.0
B factor, Ligand (Å^2)		35.4
B factor, water molecules (Å^2)	27.3	45.6
Ramachandran plot (%)		
Favored (%)	98.27	97.55
Allowed (%)	1.73	2.45
Outliers (%)	0.00	0.00
PDB code	7BTM	7BSX

Supplementary Table 4. NMR spectroscopic data for **18.**

Position	δC, type	δH (mult., *J* in Hz)
1	51.67, CH	4.27 (br s, 1H)
3	49.88, CH	3.34 (m, 1H, 9.0)
4	23.80, CH_2	2.73 (dd, 1H, 2.3, 17.2)
		2.13 (m, 1H)
5	187.56, C	
6	130.68, C	
6-M	8.30, CH_3	1.94 (s, 3H)
7	155.49, C	
7-OM	61.39, CH_3	3.88 (s, 3H, overlap)
8	181.91, C	
9	137.79, C	
10	141.99, C	
11	61.25, CH	3.88(m, 1H, overlap)
13	60.69, CH	4.04 (dd, 1H, 3.0, 6.7)
14	27.40, CH_2	2.17 (m, 1H)
		1.70 (m, 1H)
15	36.04, CH	2.65 (m, 1H)
16	62.71, CH_2	3.62 (m, 1H, overlap)
		3.55 (dd, 1H, 3.7)
17	78.60, CH	4.86 (d, 1H, 3.2)
18	68.62, CH_2	3.75 (dd, 1H, 2.6)
		3.65 (m, 1H, overlap)
1’	97.06, CH	5.05 (d, 1H, 4.6)
2’	37.86, CH_2	2.02(dd, 1H, 4.8, 14.9)
		1.88 (d, 1H, 14.8)
3’	66.97, C	
3’-M	28.60, CH_3	1.29 (s, 3H)
4’	69.91, CH	3.14 (br s, 1H)
4’-NM	46.99, CH_3	3.03 (s, 3H)
4’-NM	41.68, CH_3	3.03 (s, 3H)
5’	62.16, CH	3.95(dd, 1H, 1.8, 7.3)
6’	17.19, CH_3	1.44 (d, 3H, 7.2)

In D_2O, 500 MHz for ^1H and 125 MHz for ^{13}C NMR; chemical shifts are reported in ppm. All signals are determined by ^1H -^1H COSY, HSQC and HMBC correlation.

Supplementary Table 5. NMR spectroscopic data for **19.**

Position	δC, type	δH (muti., *J* in Hz)
1	56.06, CH	4.08 (br s, 1H)
3	55,62, CH	2.91 (d, 1H, 10.5)
4	24.88, CH_2	2.67 (m, 1H, overlap)
		2.14 (m, 1H)
5	186.00, C	
6	128.66, C	
6-M	8.75, CH_3	1.94 (s, 3H, overlap)
7	155.66, C	
7-OM	61.03, CH_3	3.97 (s, 3H, overlap)
8	181.48, C	
9	137.88, C	
10	140.80, C	
11	58.38, CH	3.66 (m, 1H)
13	61.83, CH	3.86 (br s, 1H)
14	30.67, CH_2	2.06 (m, 1H, overlap)
		1.95 (m, 1H, overlap)
15	37.91, CH	2.54 (m, 1H)
16	63.94, CH_2	3.66 (m, 1H, overlap)
		3.61 (m, 1H)
17	54.27, CH_2	3.09 (d, 1H, 11.0)
		2.87 (d, 1H, 11.6)
18	68.16, CH_2	3.79 (dd, 1H, 3.4, 10.4)
		3.54 (dd, 1H, 10.3)
1’	98.34, CH	4.89 (d, 1H, 4.6)
2’	41.01, CH_2	1.83 (dd, 1H, 4.7, 14.2)
		1.72 (d, 1H, 14.2)
3’	65.99, C	
3’-M	29.01, CH_3	1.18 (s, 3H)
4’	69.21, CH	2.06 (br s, 1H, overlap)
4’-NM	44.72, CH_3	2.65 (s, 6H, overlap)
5’	65.35, CH	3.96 (m, 1H, overlap)
6’	18.87, CH_3	1.31 (d, 3H, 7.0)

In $CDCl_3$, 600 MHz for ^1H and 150 MHz for ^{13}C NMR and DEPT135; chemical shifts are reported in ppm. All signals are determined by ^1H -^1H COSY, HSQC and HMBC correlation.

Supplementary Table 6. Information of homologous proteins selected by blastP in this study.

**Name**	**Accession**	**Origin**	**Identity**
NapW	WP_121719702.1	*S. lusitanus* NRRL 8034	100%
homW	MT230905	*S. lusitanus* NRRL 8034	76%
SfmO1	ABI22118.1	*S. lavendulae* NRRL11002	56%
homO1a	MT230906	*S. lavendulae* NRRL11002	62%
homO1b	MT230907	*S. lavendulae* NRRL11002	60%
SlvW1	WP_003978122.1	*S. lividans* 1326	74%
SlvW2	EOY50906.1	*S. lividans* 1326	61%
SDR-Pb	WP_076164526.1	*Paenibacillus*	75%
SDR-Cc	WP_099700661.1	*Chroococcales cyanobacterium*	67%
SDR-Rm	WP_028745990.1	*Rhizobium mesoamericanum*	65%
SDR-Mb	OJY30843.1	Myxococcales bacterium	53%
SDR-Li	WP_002092909.1	*Leptospira interrogans*	49%
SDR-Cs	PRW56829.1	*Chlorella sorokiniana*	44%
SDR-Ss	WP_094604007.1	*Sporomusa silvacetica*	34%

Supplementary Figures

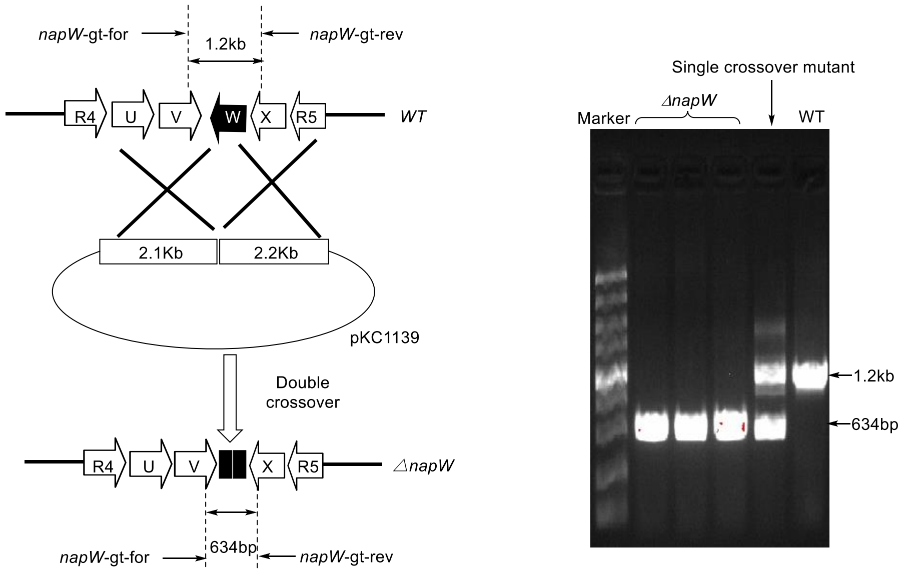

Supplementary Figure 1. Construction and verification of *napW* gene-knockout mutant (*ΔnapW*, TG3022) of *S. lusitanus* NRRL 8034. **a,** Double crossover process. **b,** Genotyping validation of three *ΔnapW* mutant clones. One single crossover mutant clone and wild type (WT) were performed as negative control.

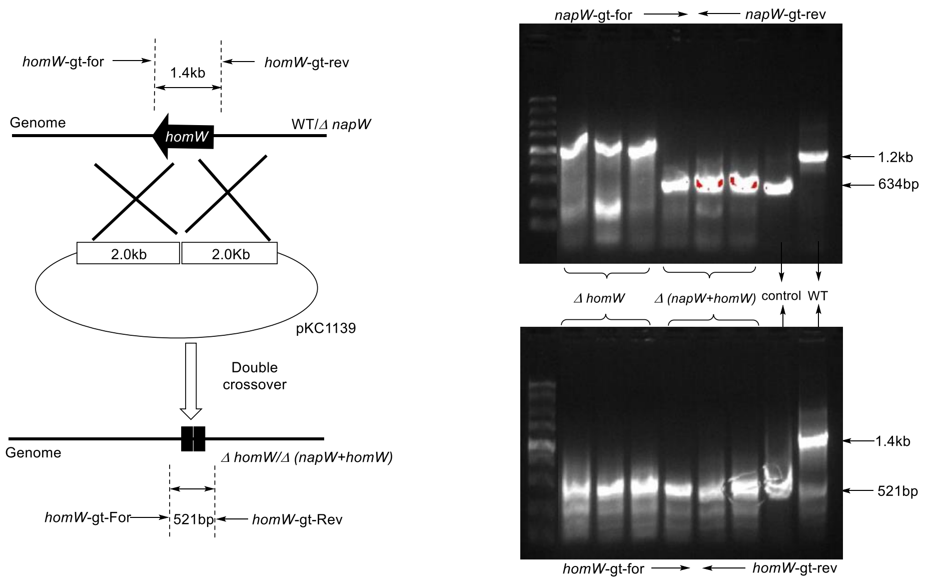

Supplementary Figure 2. Construction and verification of *homW* (*ΔhomW*, TG3023) and dual-gene (*ΔnapW&ΔhomW,* TG3024) gene-knockout mutants of *S. lusitanus* NRRL 8034. **a,** Double crossover process. **b,** Genotyping validation of three *ΔhomW* mutant clones and three *ΔnapW&ΔhomW* mutant clones. Control panels were plasmids pTG3032 and pTG3046 as positive control. Wild type (WT) was performed as negative control.

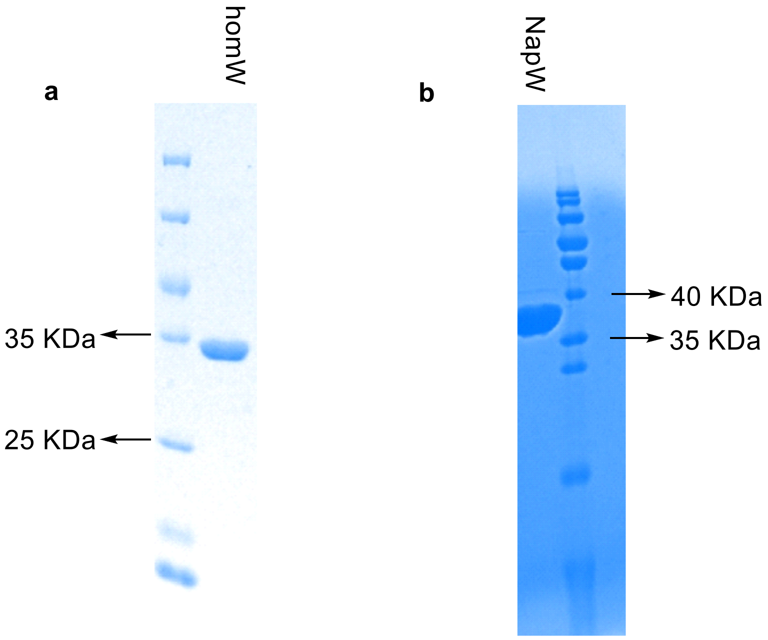

Supplementary Figure 3. Overproduction in *E. coli* and purification of two enzymes utilized in this study. **a,** SDS-PAGE analysis of purified homW. **b,** SDS-PAGE analysis of purified NapW.

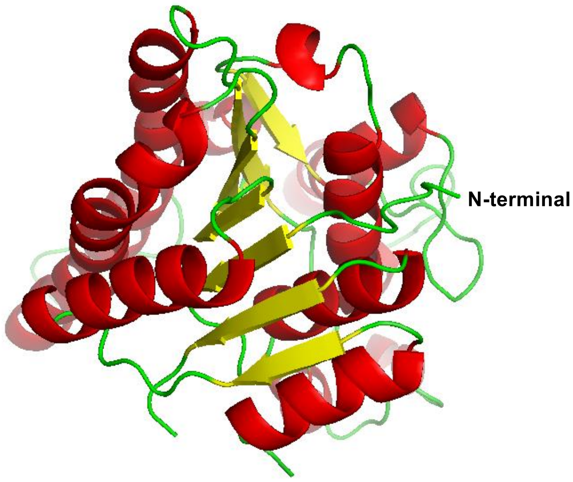

Supplementary Figure 4. Cartoon representation of chain A of NapW structure.

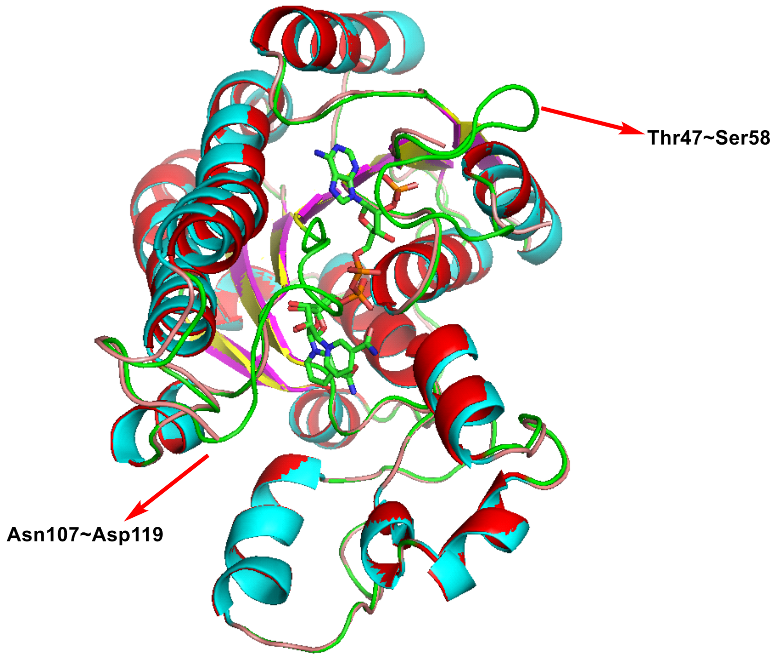

Supplementary Figure 5. Overlapping for structures of NapW-NADPH complex (helix/sheet/loop, red/yellow/green) and NapW (helix/sheet/loop, cyan/magenta/tint).

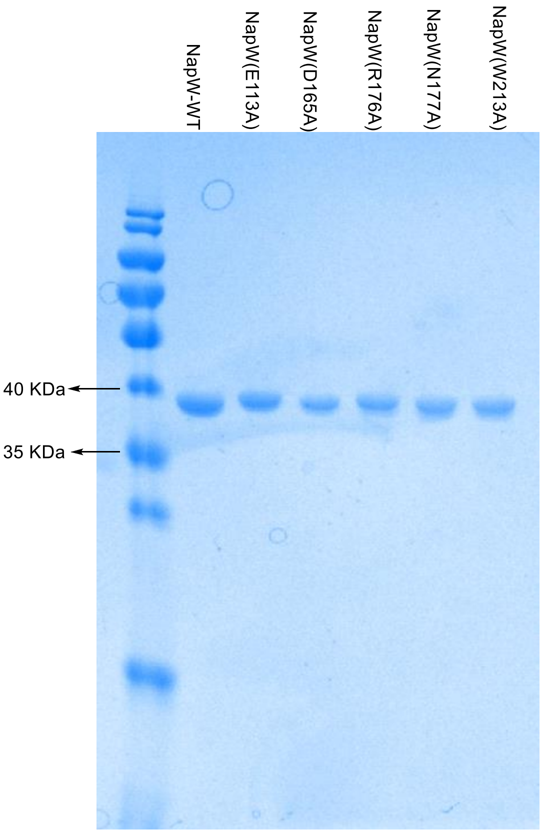

Supplementary Figure 6. Overproduction in *E. coli* and purification of NapW and mutants utilized in this study. These proteins were analyzed by SDS-PAGE.

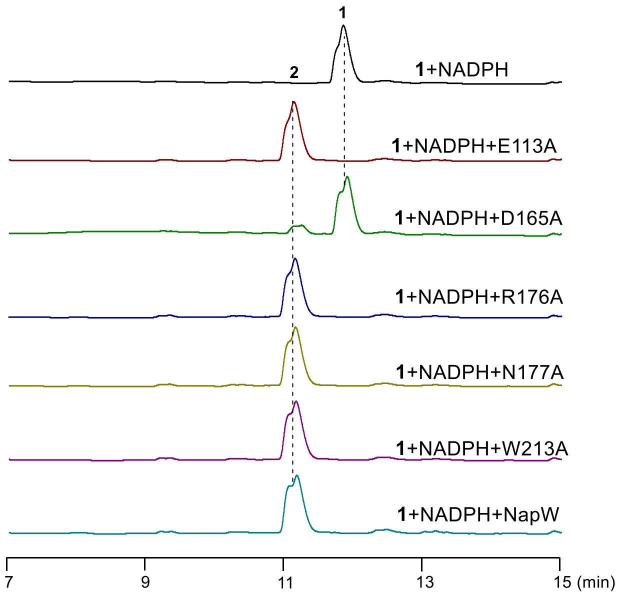

Supplementary Figure 7. Catalytic activity of the NapW mutants to NDM detected by HPLC. The HPLC analysis with UV detected at 270 nm.

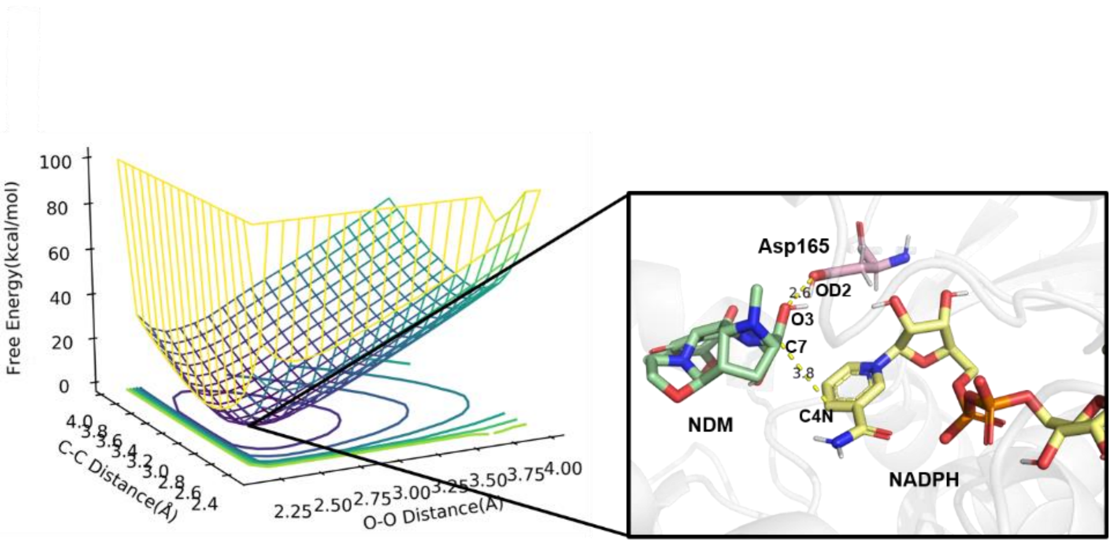

Supplementary Figure 8. The potential energy surface obtained from umbrella sampling. The d(OD2-O3) represents the distance between the OD2 of Asp165 and O3 of NDM. The d(C7-C4N) represents the distance between the C7 of NDM and the C4N of NADPH.

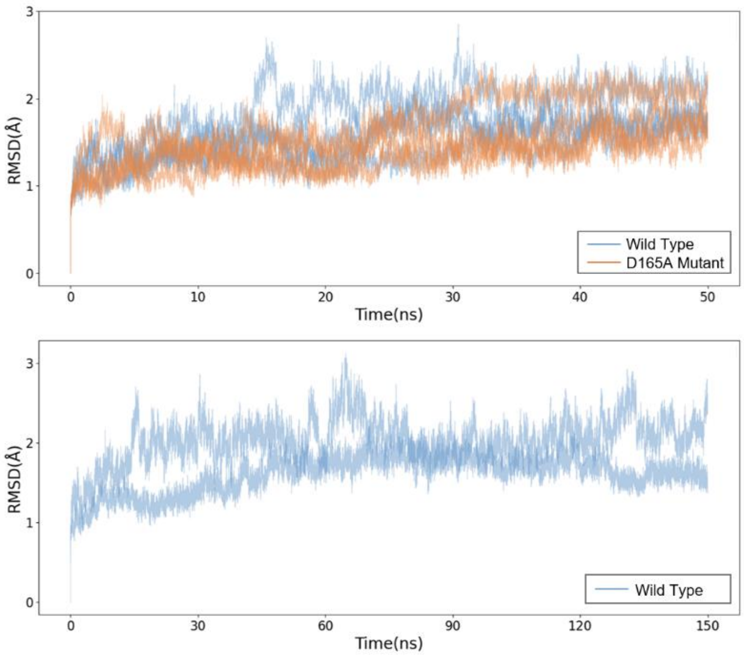

Supplementary Figure 9. The RMSD values of MD simulations in WT and D165A mutant systems. The RMSD values of 4×50 ns MD simulations in wild type and D165A mutant systems respectively. The wild type system was colored in blue while D165A mutant system was colored in orange. The RMSD values of 2×150 ns MD simulations in wild type system were demonstrated in the bottom.

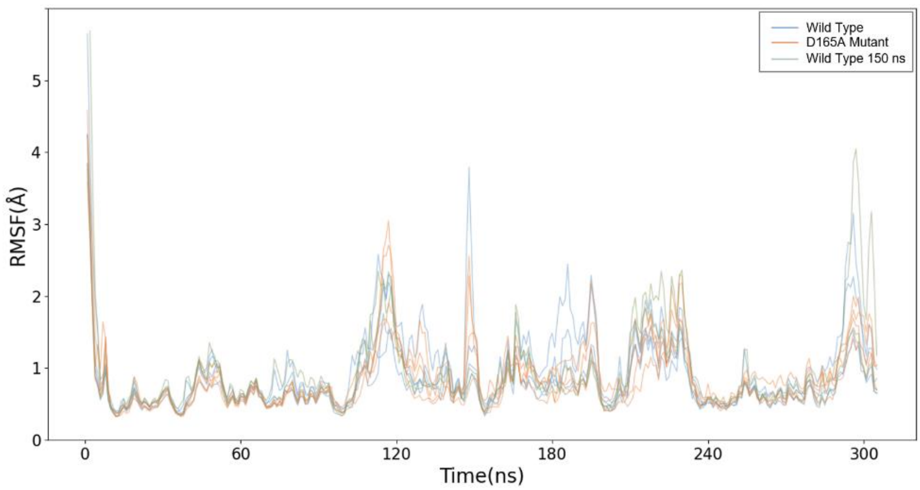

Supplementary Figure 10. The RMSF values of MD simulations in WT and D165A mutant. The RMSF values of 4×50 ns MD simulations in wild type system and D165A mutant systems were shown in blue and orange respectively. The RMSF values of 2×150 ns MD simulations in wild type system were shown in green.

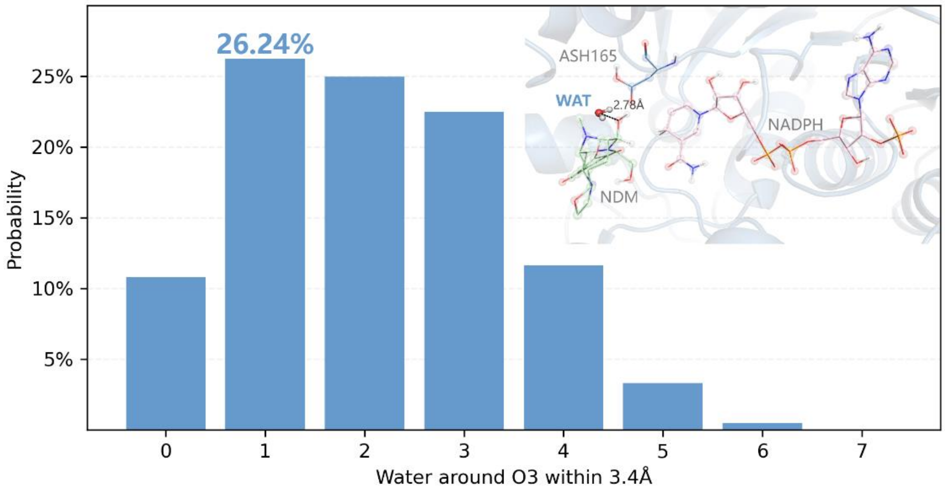

Supplementary Figure 11. The probability of water number within 3.4 Å of O3 atom of NDM in MD simulations. The water number within 3.4 Å of O3 atom of NDM was counted in two 150 ns trajectories. In the upper right, the representative water molecule was shown.

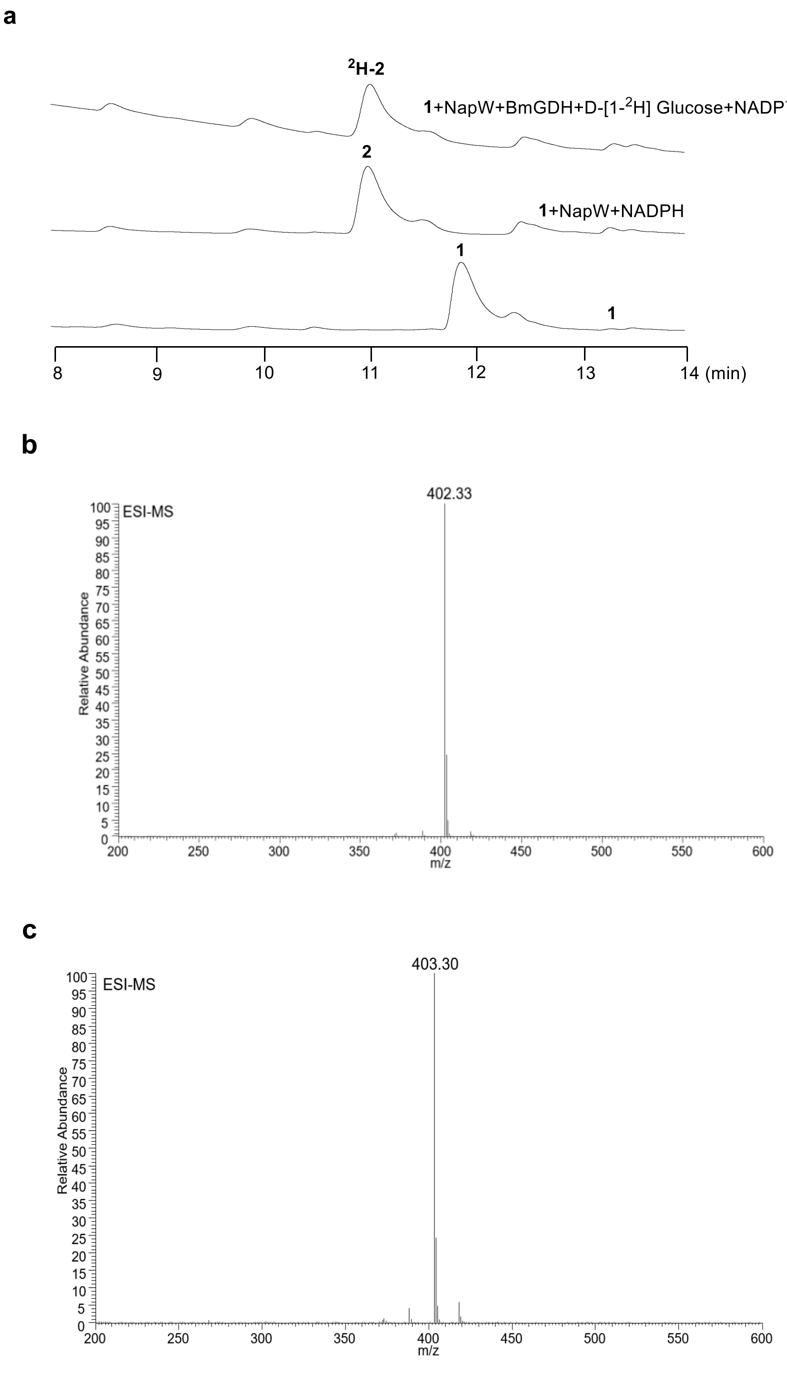

Supplementary Figure 12. Analysis of NapW/BmGDH cascade reaction. **a,** Result of NapW/BmGDH cascade reaction is detected by HPLC compared with controls, the HPLC analysis with UV detected at 270 nm. **b,** NapW-catalyzed product **2** is analyzed by ESI-MS. **c,** NapW/BmGDH-catalyzed product, ^{2}**H-2** is analyzed by ESI-MS.

a

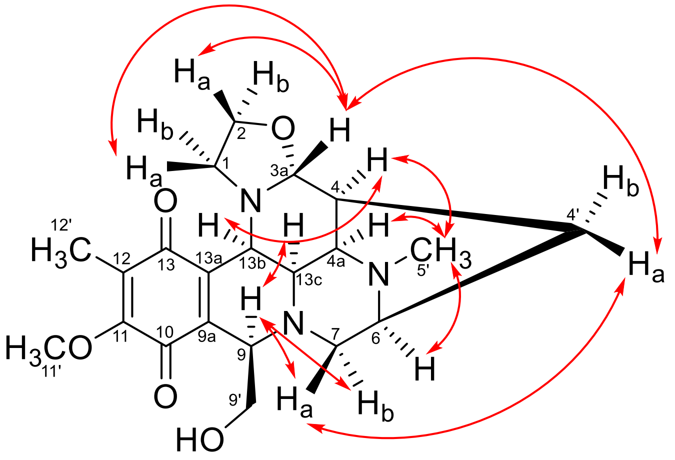

b

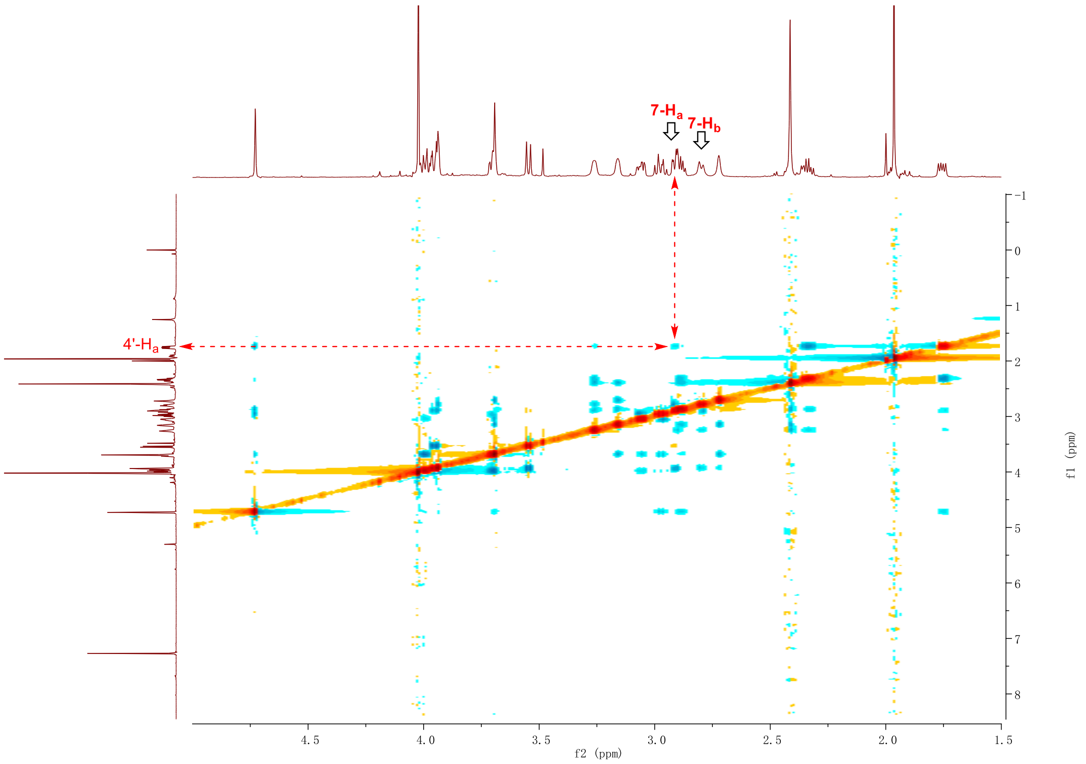

Supplementary Figure 13. a, NOEs (indicated by red arrows) determined the stereochemistry of compound **2**. **b,** The NOE (indicated by red dashed arrows) between 4'-H_a and 7-H_a indicated the positions of 7-H_a and 7-H_b in space.

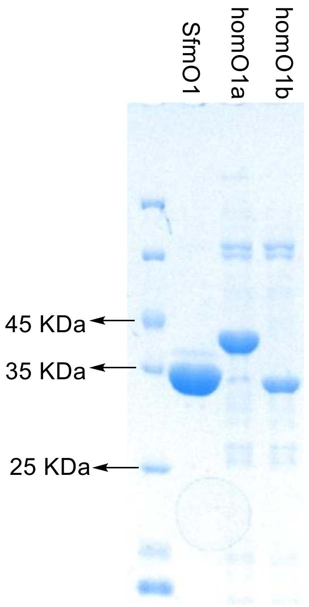

Supplementary Figure 14. Overproduction in *E. coli* and purification of SfmO1, homO1a and homO1b utilized in this study. These proteins were analyzed by SDS-PAGE.

a

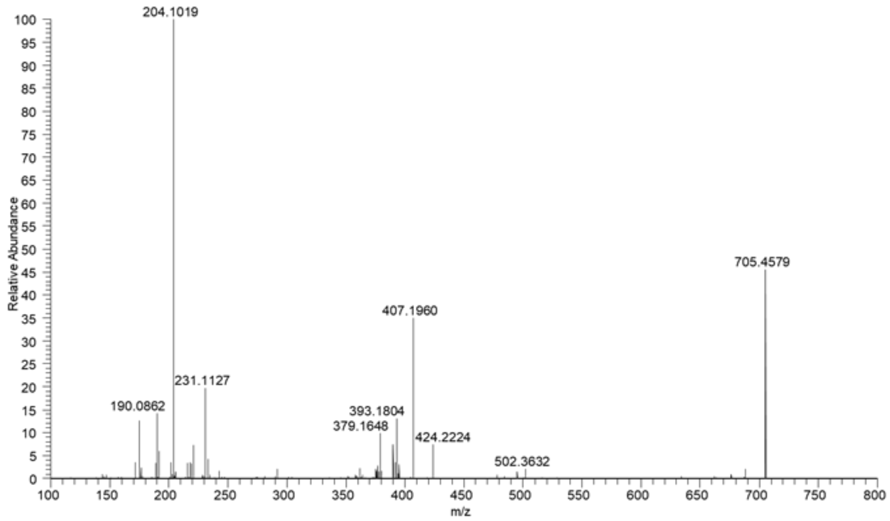

b

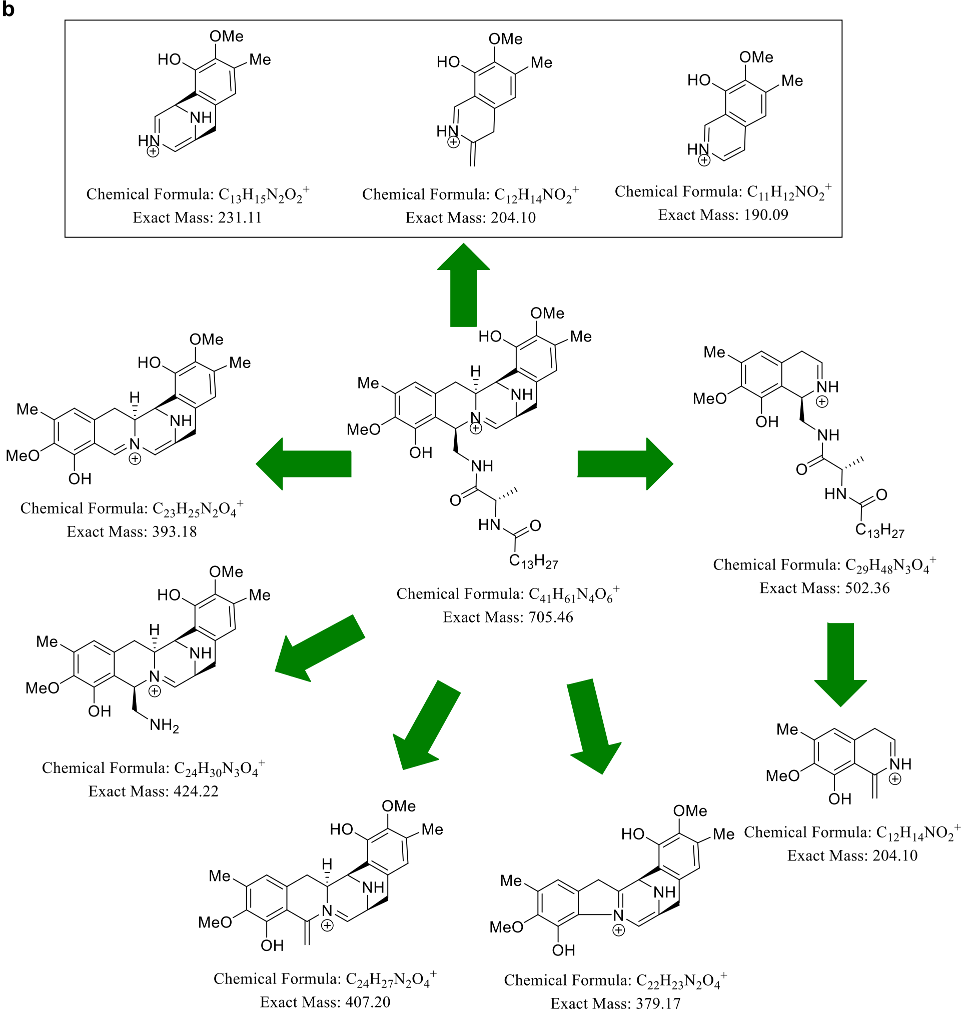

Supplementary Figure 15. HR-MS/MS analysis of **16. a,** HR-MS/MS analysis of **16**. **b,** Analysis of fragments of **16**.

a

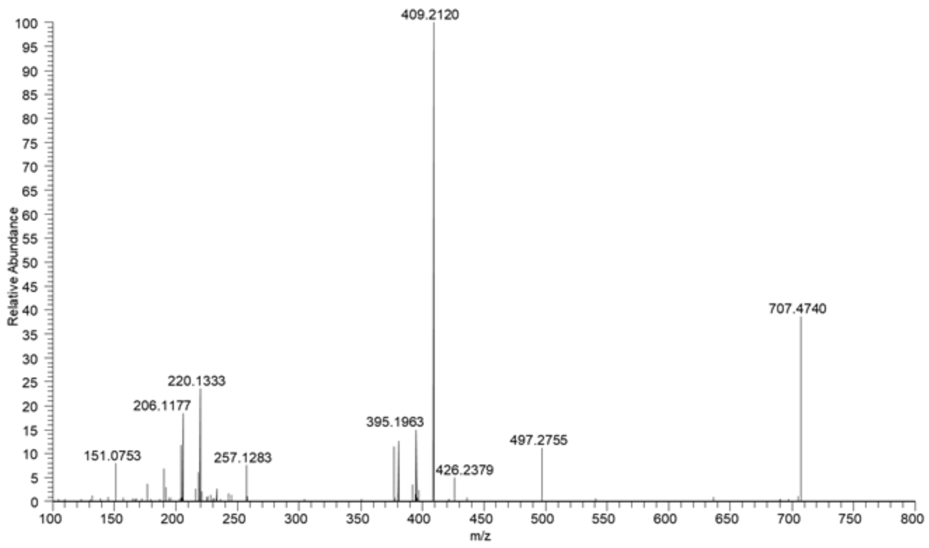

b

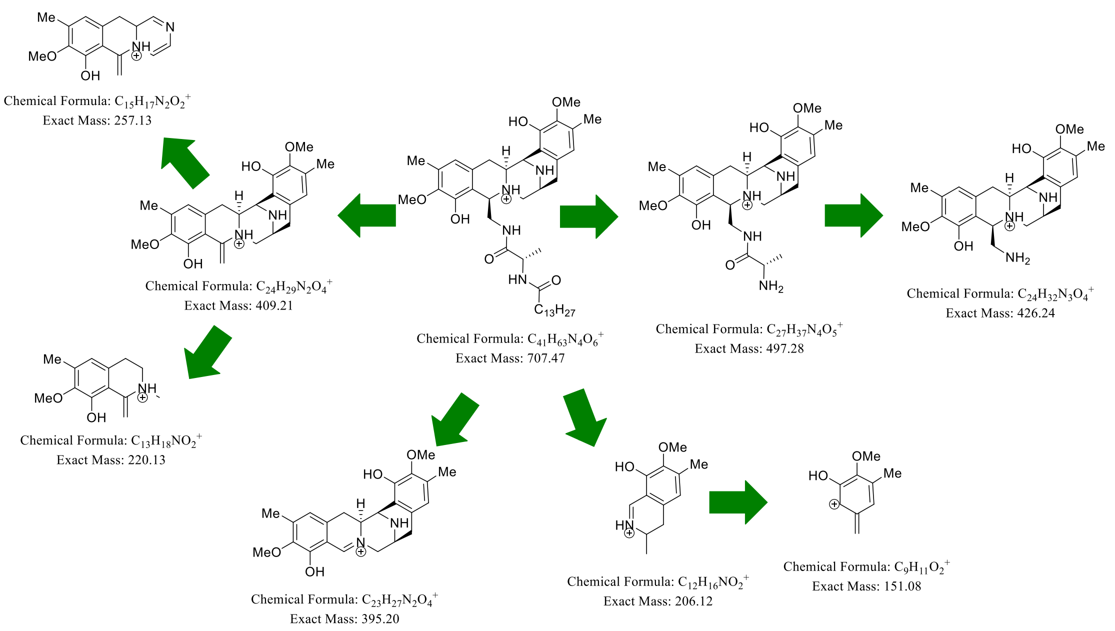

Supplementary Figure 16. HR-MS/MS analysis of **17**. **a,** HR-MS/MS analysis of **17**. **b,** Analysis of fragments of **17**.

a

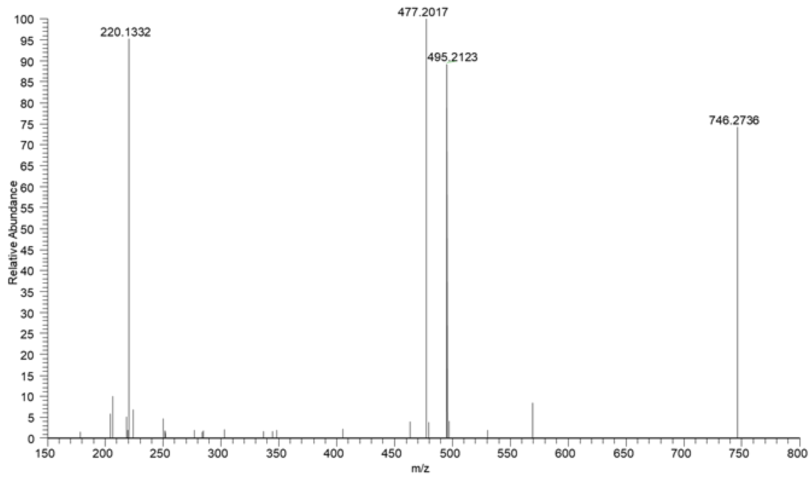

b

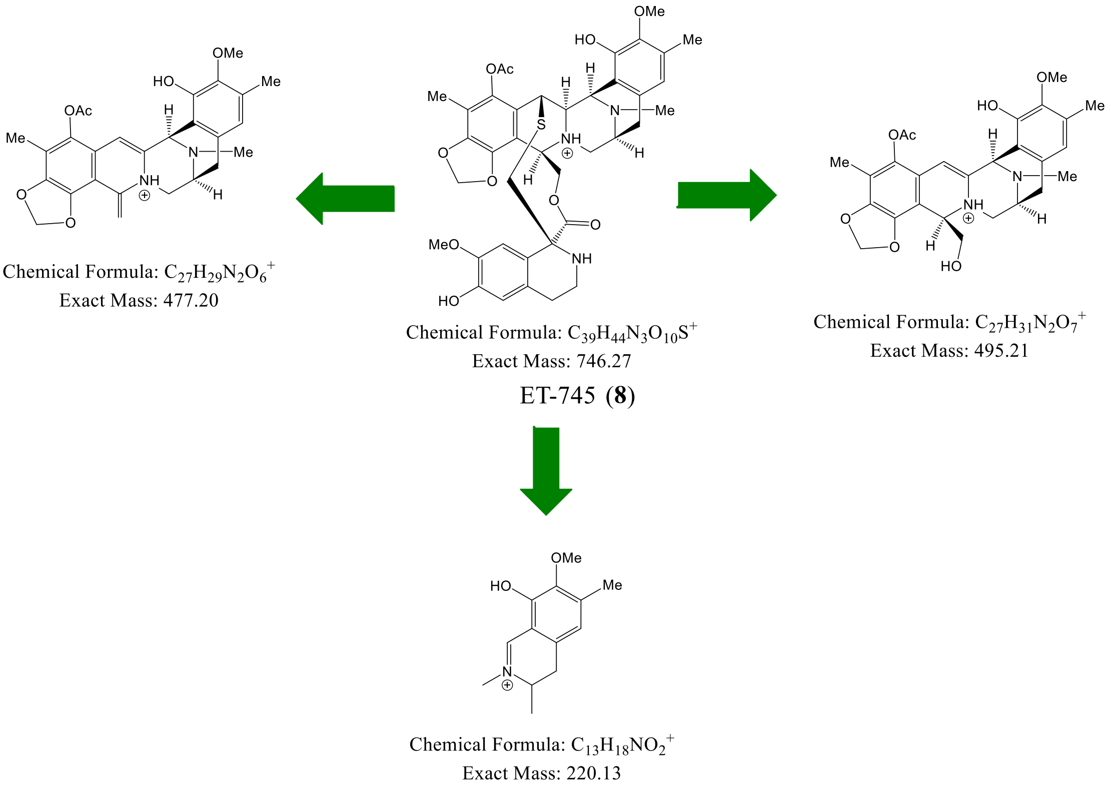

Supplementary Figure 17. HR-MS/MS analysis of ET-745 (**8**). **a,** HR-MS/MS analysis of **8**. **b,** Analysis of fragments of **8**.

a

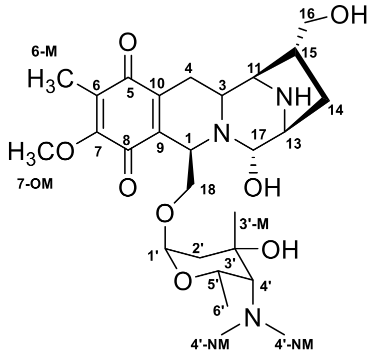

18

b

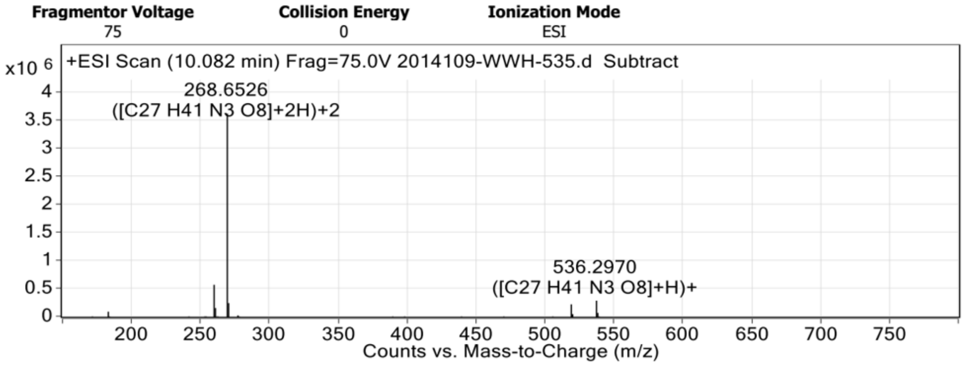

Formula Calculator Results

Ion Formula	m/z	m/z (Calc)	Diff (ppm)	DBE	Score (MFG)
C27 H42 N3 O8	536.2970	536.2966	-0.67	9	99.76
C30 H40 N4 O5	536.2970	536.2993	4.34	13.5	90.76
C39 H38 N O	536.2970	536.2948	-4.13	22	91.55

Ion Formula	m/z	m/z (Calc)	Diff (ppm)	DBE	Score (MFG)
C27 H43 N3 O8	268.6526	268.6520	-2.39	9	96.97
C30 H41 N4 O5	268.6526	268.6533	2.61	13.5	96.41

c

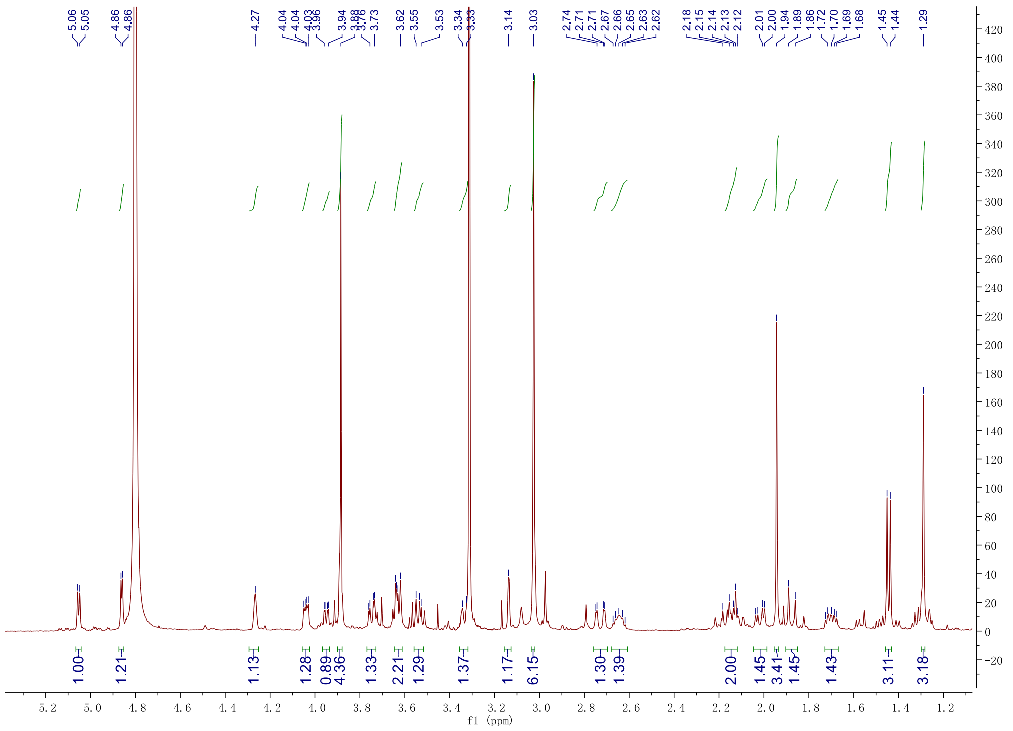

d

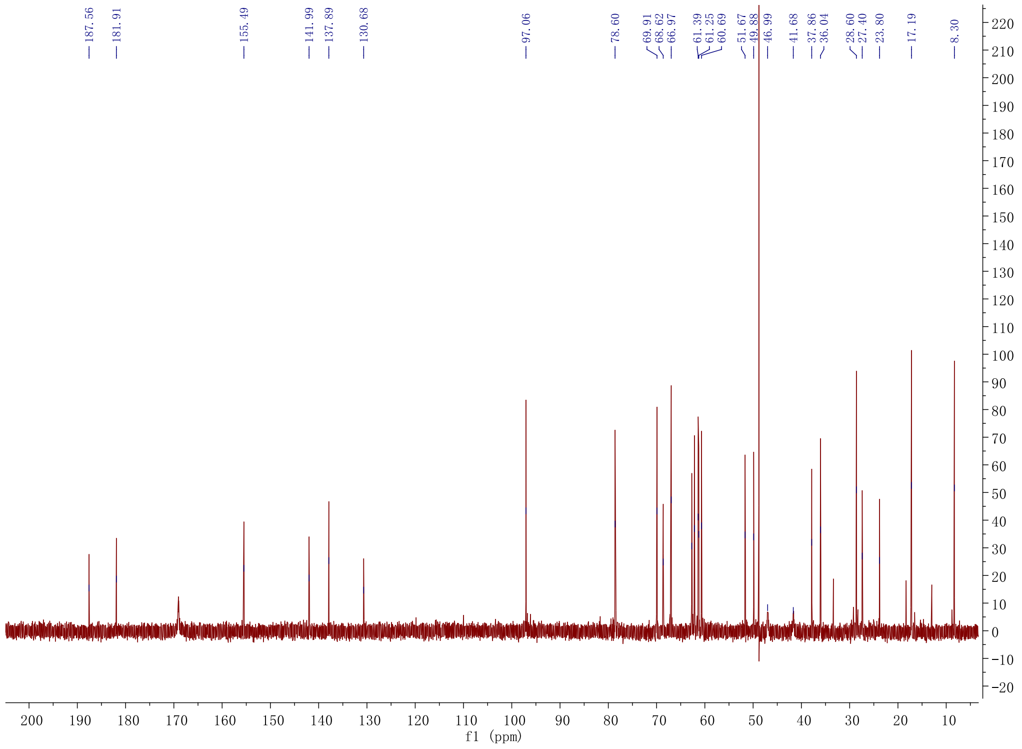

e

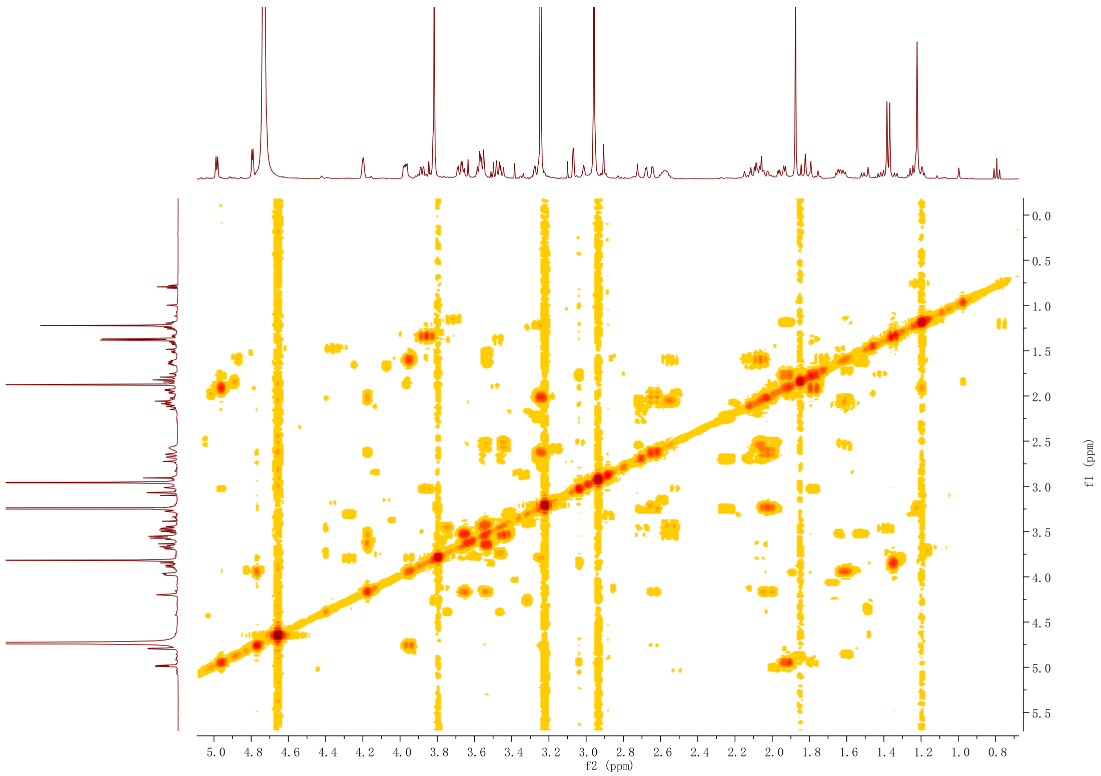

f

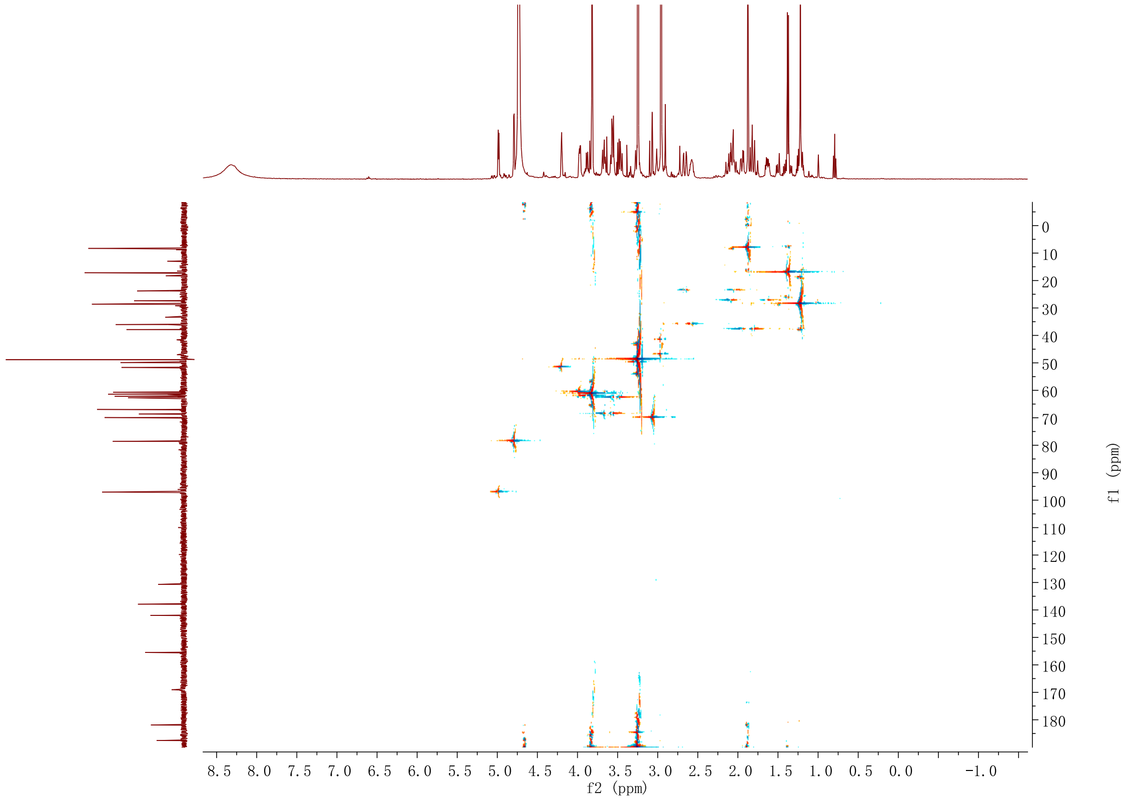

g

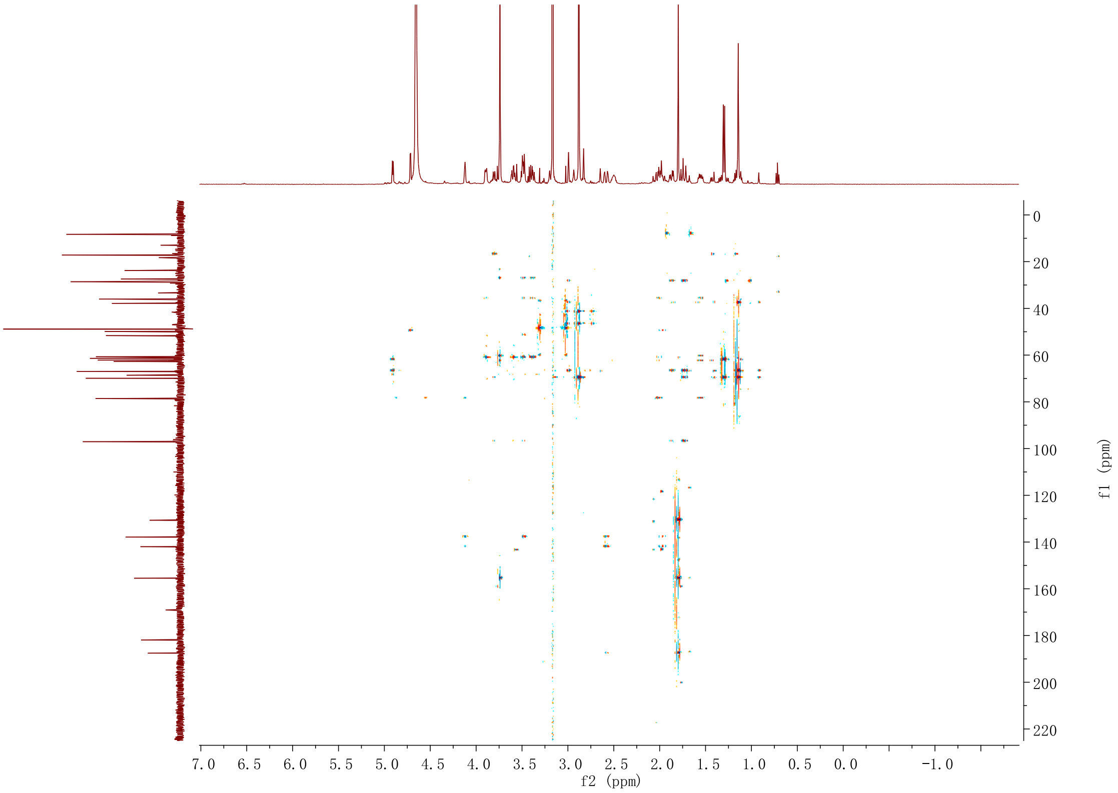

Supplementary Figure 18. Structure characterization data of **18**. The stereochemistry of **18** was determined by comparing with LMM[1]. **a,** Structure of **18**. **b,** HR-MS analysis of **18**. HRMS (ESI): m/z = 536.2970 ($[M+H]^+$), m/z (calculated [calc.]) = 536.2966 ($[M+H]^+$) consistent with the molecular formula $C_{27}H_{41}N_3O_8$; UV max: 222 nm, 272 nm. **c,** ^{1}H NMR spectrum of **18**. **d,** ^{13}C NMR spectrum of **18**. **e,** ^{1}H-^{1}H COSY spectrum of **18**. **f,** HSQC spectrum of **18**. **g,** HMBC spectrum of **18**.

a

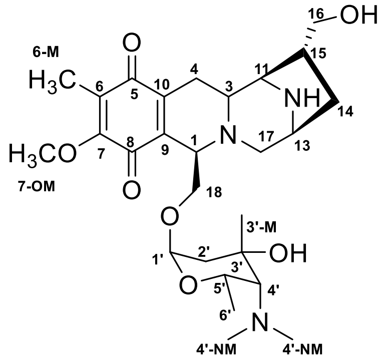

19

b

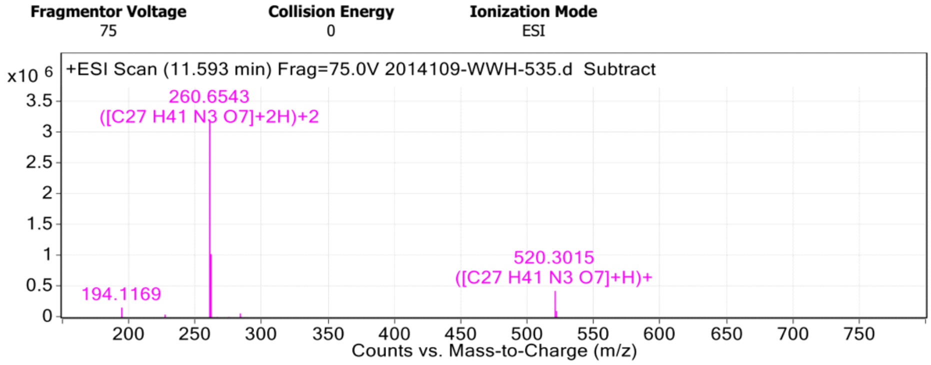

Formula Calculator Results

Ion Formula	m/z	m/z (Calc)	Diff (ppm)	DBE	Score (MFG)
C24 H45 N2 O10	260.6543	260.6532	-4.38	4.5	90.78
C27 H43 N3 O7	260.6543	260.6545	0.78	9	99.68

Ion Formula	m/z	m/z (Calc)	Diff (ppm)	DBE	Score (MFG)
C24 H44 N2 O10	520.3015	520.2990	-4.72	4.5	89.47
C27 H42 N3 O7	520.3015	520.3017	0.44	9	99.9

c

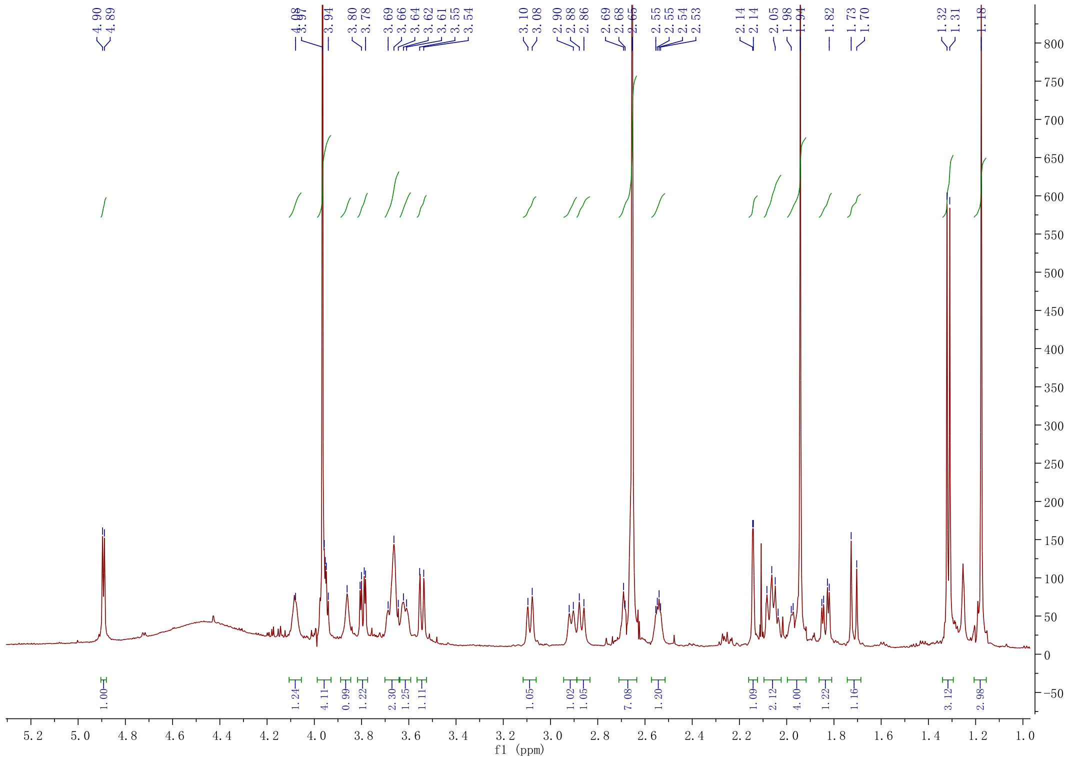

4.90
4.89
3.97
3.94
3.80
3.78
3.69
3.66
3.64
3.62
3.61
3.55
3.54
3.10
3.08
2.90
2.88
2.86
2.69
2.68
2.55
2.55
2.54
2.53
2.14
2.14
2.05
1.98
1.82
1.73
1.70
1.32
1.31
1.00
1.24
4.11
0.99
1.22
2.30
1.25
1.11
1.05
1.02
1.05
7.08
1.20
1.09
2.12
4.00
1.22
1.16
3.12
2.98
f1 (ppm)

d

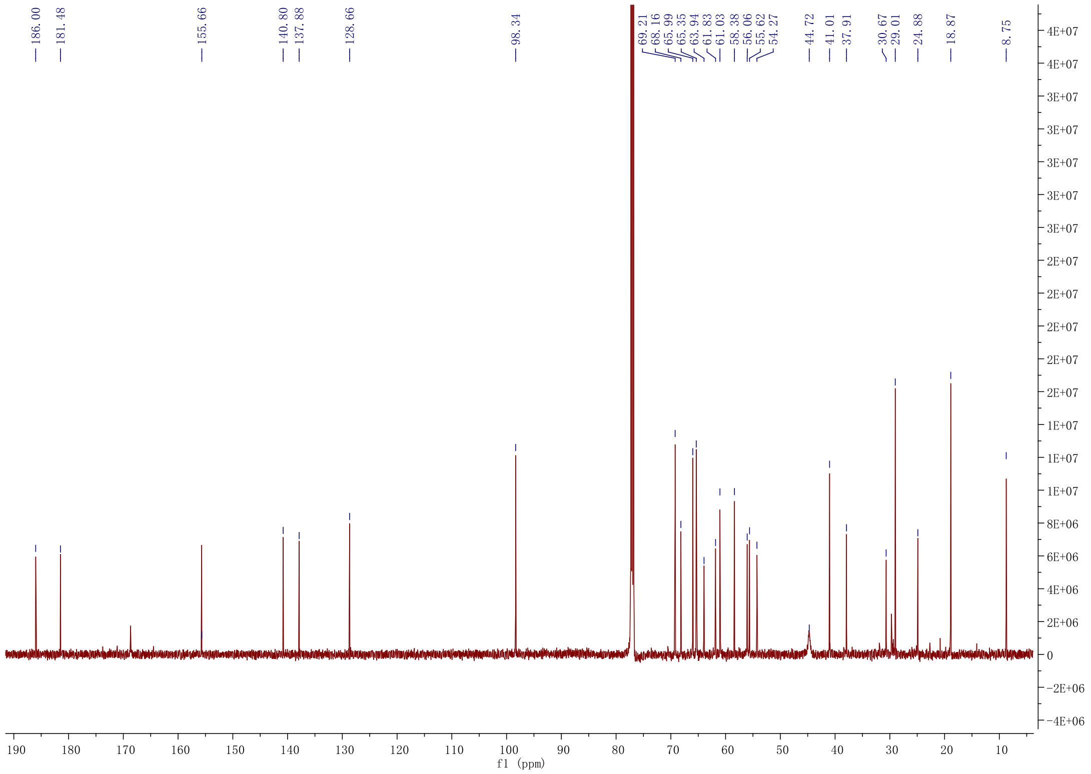

186.00
181.48
155.66
140.80
137.88
128.66
98.34
69.21
68.16
65.99
65.35
63.94
61.83
61.03
58.38
56.06
55.62
54.27
44.72
41.01
37.91
30.67
29.01
24.88
18.87
8.75
f1 (ppm)

e

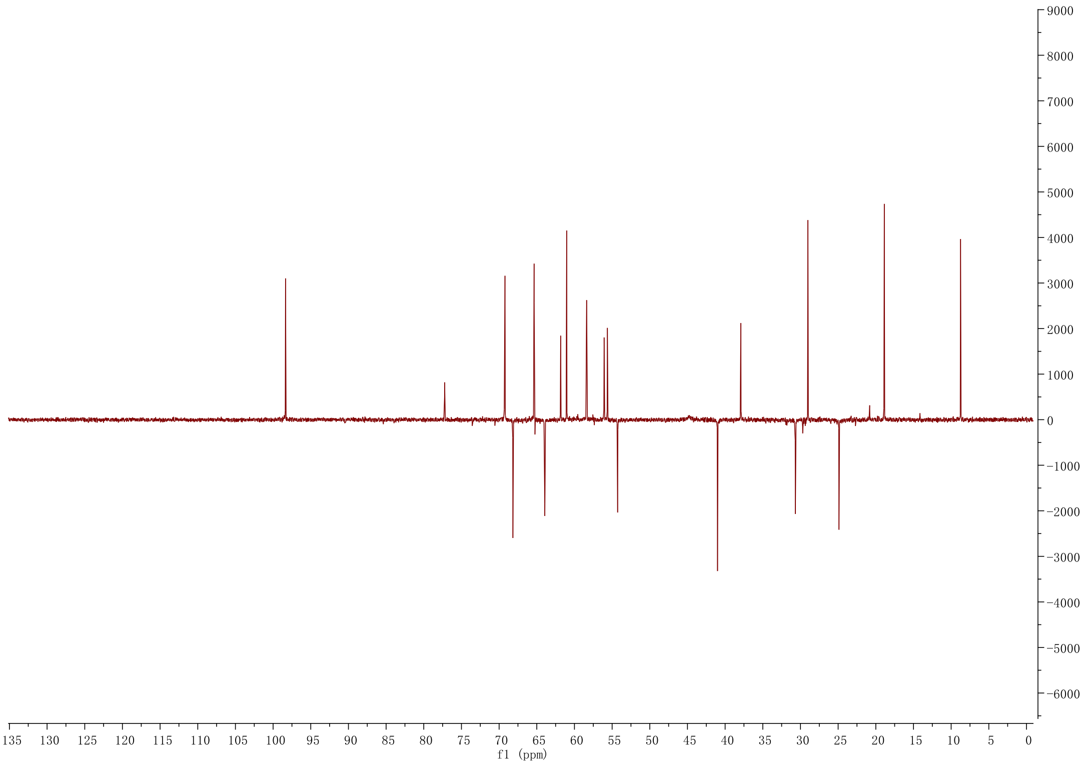

9000
8000
7000
6000
5000
4000
3000
2000
1000
0
−1000
−2000
−3000
−4000
−5000
−6000
135 130 125 120 115 110 105 100 95 90 85 80 75 70 65 60 55 50 45 40 35 30 25 20 15 10 5 0
f1 (ppm)

f

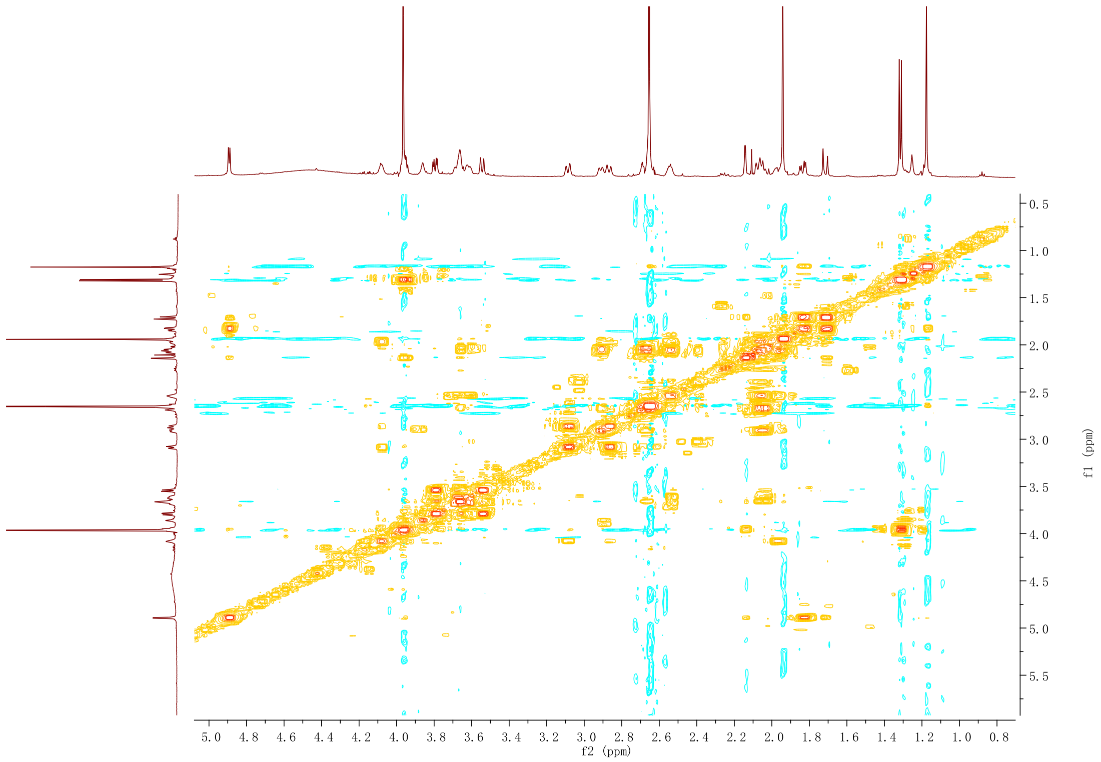

0.5 1.0 1.5 2.0 2.5 3.0 3.5 4.0 4.5 5.0 5.5
f1 (ppm)
5.0 4.8 4.6 4.4 4.2 4.0 3.8 3.6 3.4 3.2 3.0 2.8 2.6 2.4 2.2 2.0 1.8 1.6 1.4 1.2 1.0 0.8
f2 (ppm)

g

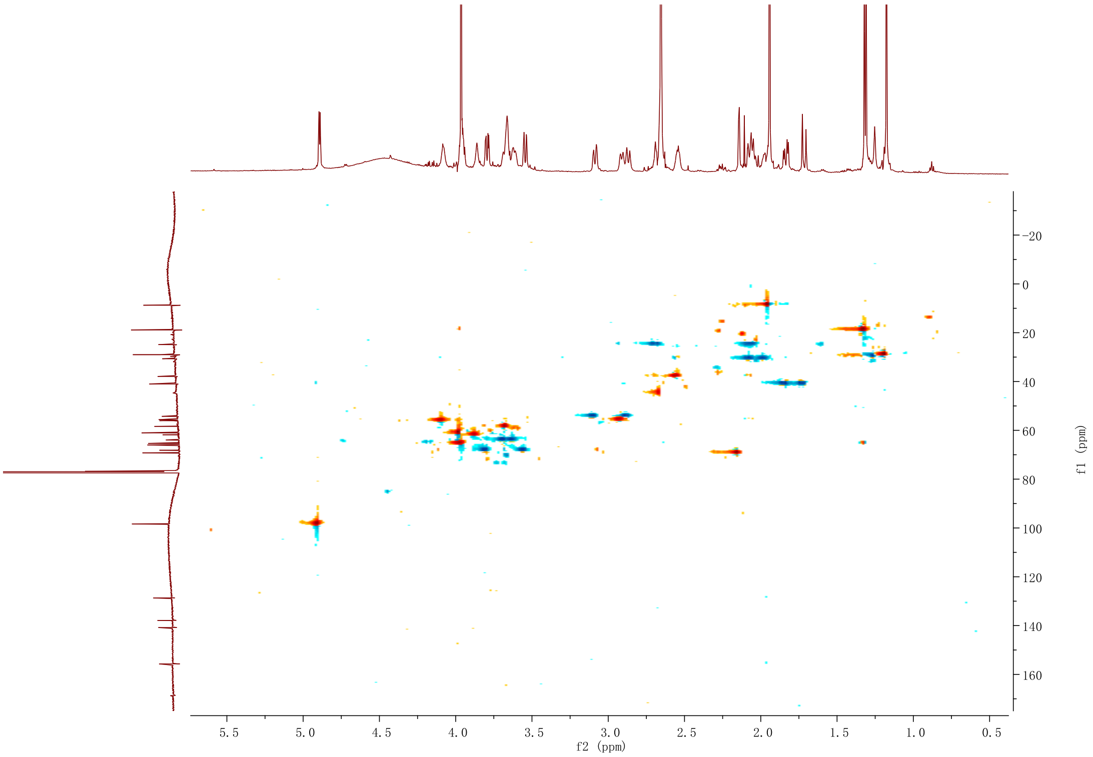

h

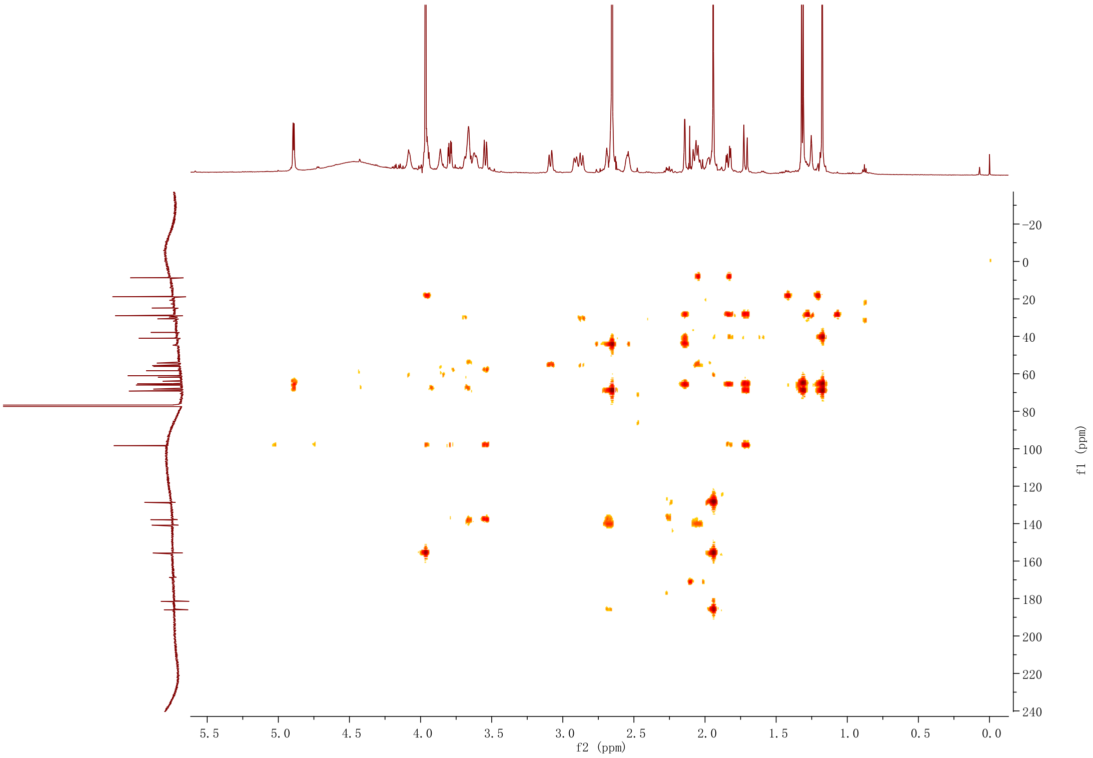

Supplementary Figure 19. Structure characterization data of **19**. **a,** Structure of **19**. **b,** HR-MS analysis of **19**. HRMS (ESI): m/z = 520.3015 ([M+H]$^+$), m/z (calculated [calc.]) = 520.3017 ([M+H]$^+$) consistent with the molecular formula $C_{27}H_{41}N_3O_7$; UV max: 222 nm, 272 nm. **c,** ^{1}H NMR spectrum of **19**. **d,** ^{13}C NMR spectrum of **19**. **e,** DEPT135

spectrum of **19**. **f,** ^{1}H-^{1}H COSY spectrum of **19**. **g,** HSQC spectrum of **19**. **h,** HMBC spectrum of **19.**

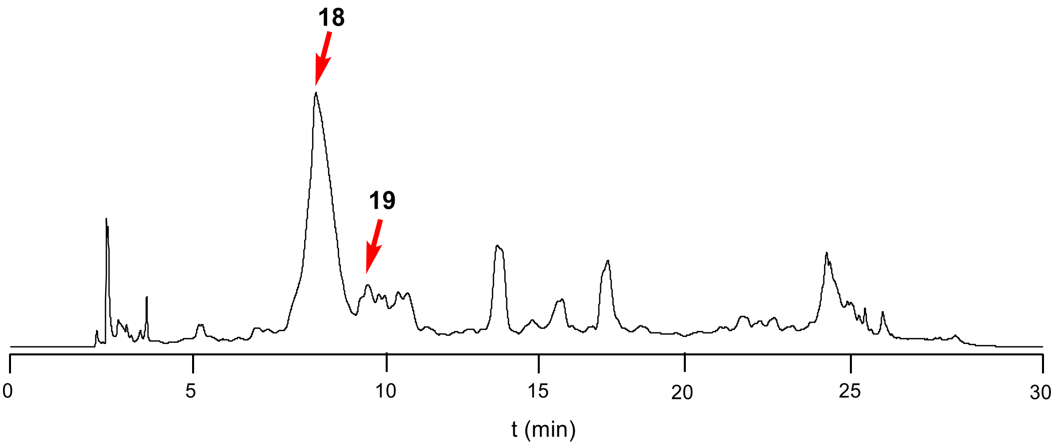

Supplementary Figure 20. Fermentation analysis of LMM producing strain, *S. candidus* LL-AP191. The HPLC analysis with UV detected at 270 nm. The peaks of **18** and **19** both were detected in fermentation supernatant.

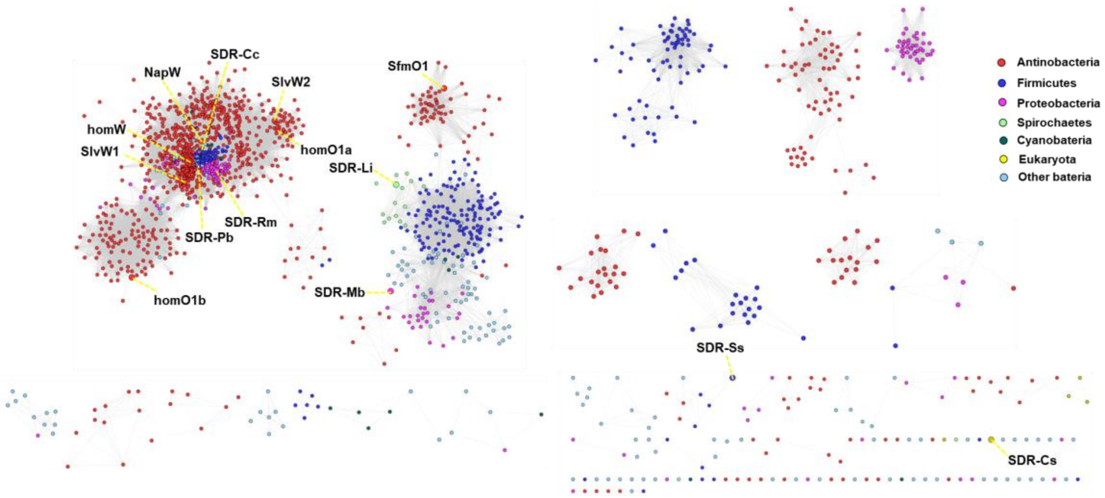

Supplementary Figure 21. Sequence similarity network (SSN) analysis of NapW-homologues. 4771 homologous proteins of NapW (above 34% sequence identity with NapW) were clustered by sequence identity cut-off[2] at 0.9. The SSN was generated from the representatives of the clusters and NapW, homW, SfmO1, homO1a, homO1b by using the online Enzyme Function Initiative-Enzyme Similarity Tool[3]. Cytoscape was used to view the SSN with an alignment score threshold of 10^{-105}. The proteins mentioned in manuscript were marked.

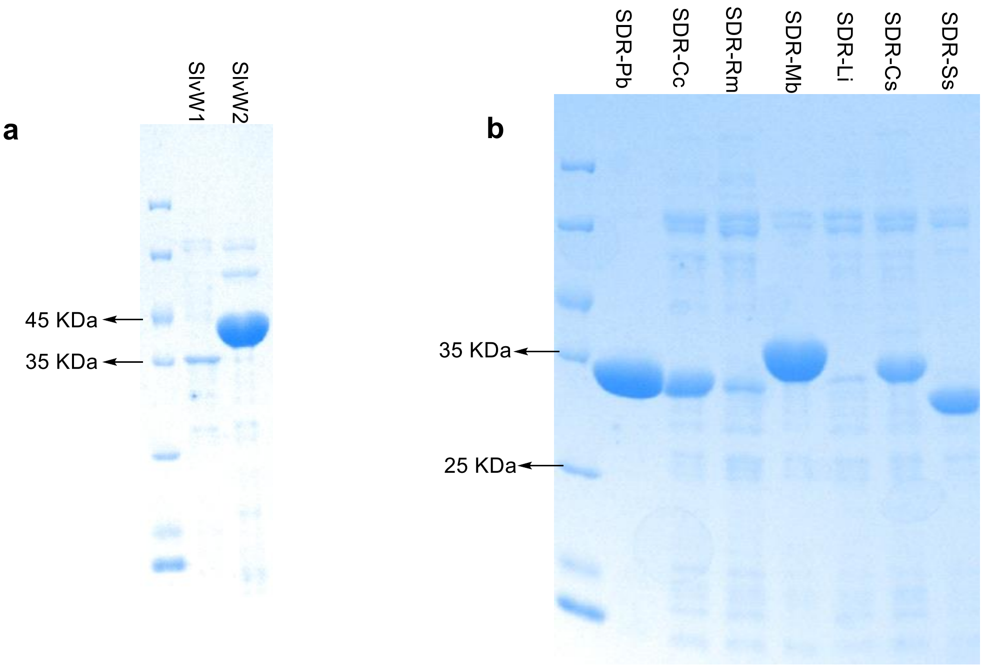

Supplementary Figure 22. Overproduction in *E. coli* and purification of SDR utilized in this study. **a,** SDS-PAGE analysis of SlvW1 and SlvW2. **b,** SDS-PAGE analysis of SDRs: SDR-Pb, SDR-Cc, SDR-Rm, SDR-Mb, SDR-Li, SDR-Cs, SDR-Ss.

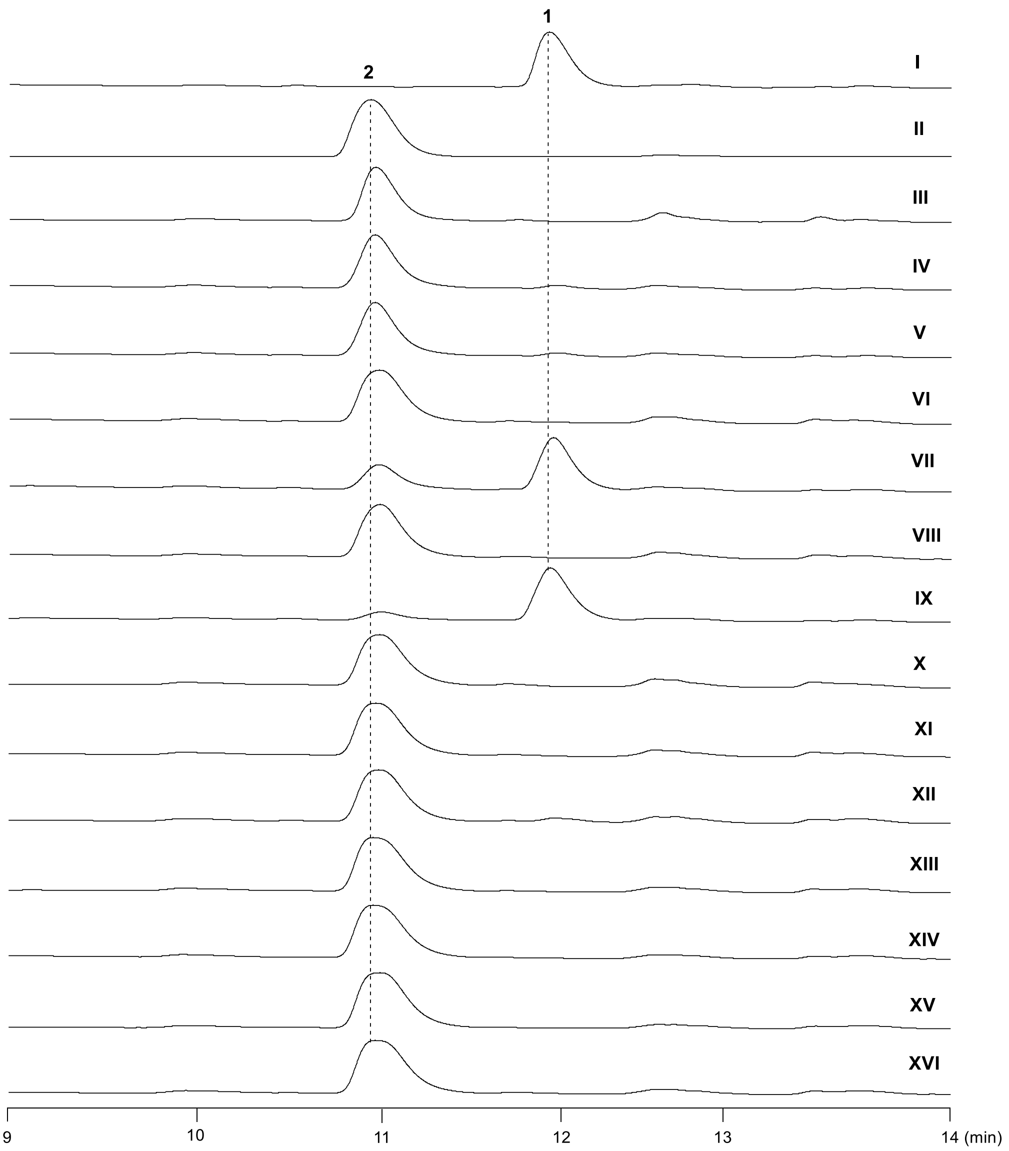

Supplementary Figure 23. Enzymatic assays of NapW and homologous proteins towards **1** were detected by HPLC analysis with UV absorption wavelength at 270 nm. (I) Substrate **1** prepared by NapU-catalyzed reaction + NADPH, (II) Standard **2**, (III) **1** + NADPH + SDR-Pb, (IV) **1** + NADPH + SDR-Cc, (V) **1** + NADPH + SDR-Rm, (VI) **1** + NADPH + SDR-Mb, (VII) **1** + NADPH+SDR-Li, (VIII) **1** + NADPH + SDR-Cs, (IX) **1** + NADPH + SDR-Ss, (X) **1** + NADPH + NapW, (XI) **1** + NADPH + homW, (XII) **1** + NADPH + SfmO1, (XIII) **1** + NADPH + homO1a, (XIV) **1** + NADPH + homO1b, (XV) **1** + NADPH + SlvW1, (XVI) **1** + NADPH + SlvW2.

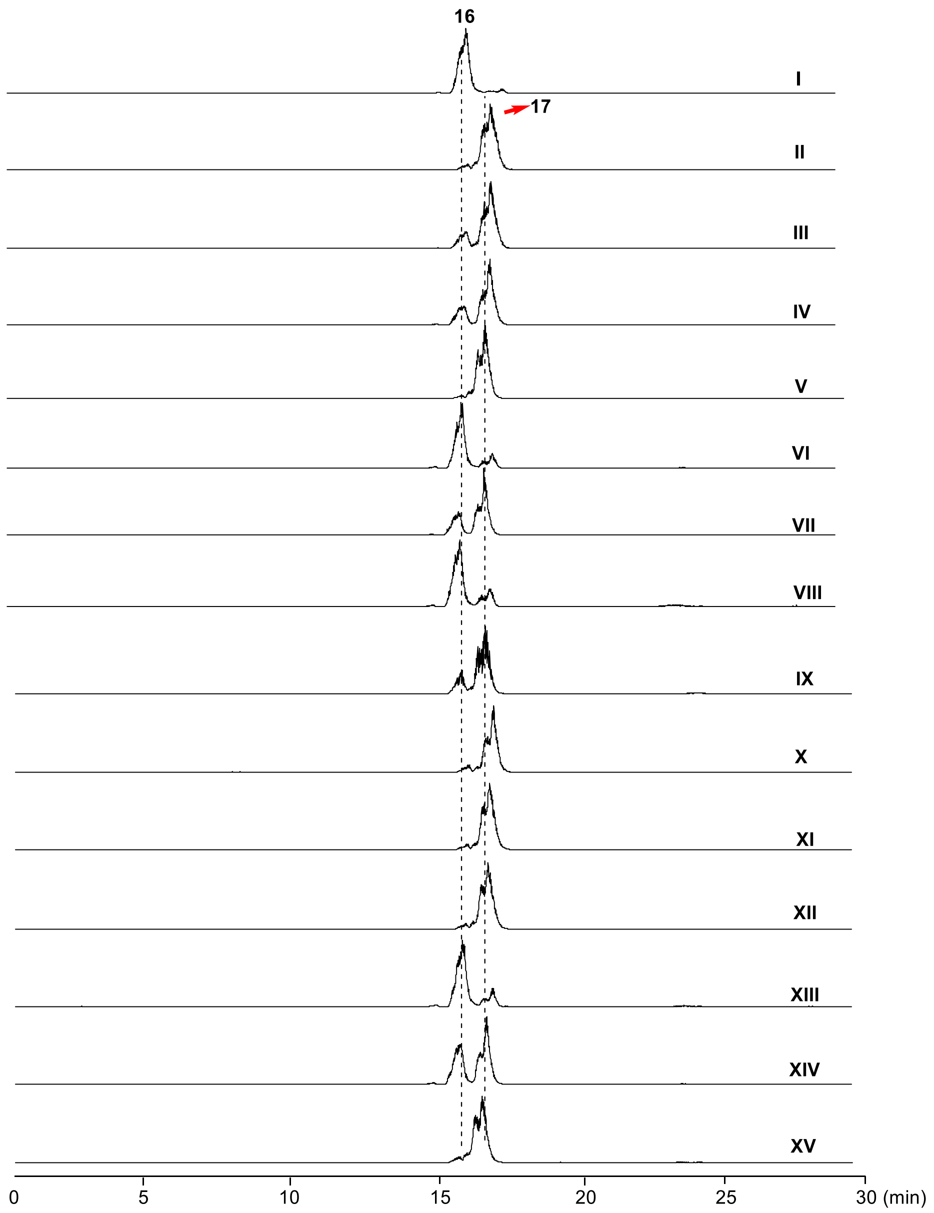

Supplementary Figure 24. Enzymatic assays of NapW and homologous proteins towards **16** were detected by HPLC-MS. (I) Substrate **16** prepared by SfmC-catalyzed reaction + NADPH, (II) **16** + NADPH + SDR-Pb, (III) **16** + NADPH + SDR-Cc, (IV) **16** + NADPH + SDR-Rm, (V) **16** + NADPH + SDR-Mb, (VI) **16** + NADPH+SDR-Li, (VII) **16** + NADPH + SDR-Cs, (VIII) **16** + NADPH + SDR-Ss, (IX) **16** + NADPH + NapW, (X) **16** + NADPH + homW, (XI) **16** + NADPH + SfmO1, (XII) **16** + NADPH + homO1a, (XIII) **16** + NADPH + homO1b, (XIV) **16** + NADPH + SlvW1, (XV) **16** + NADPH + SlvW2.

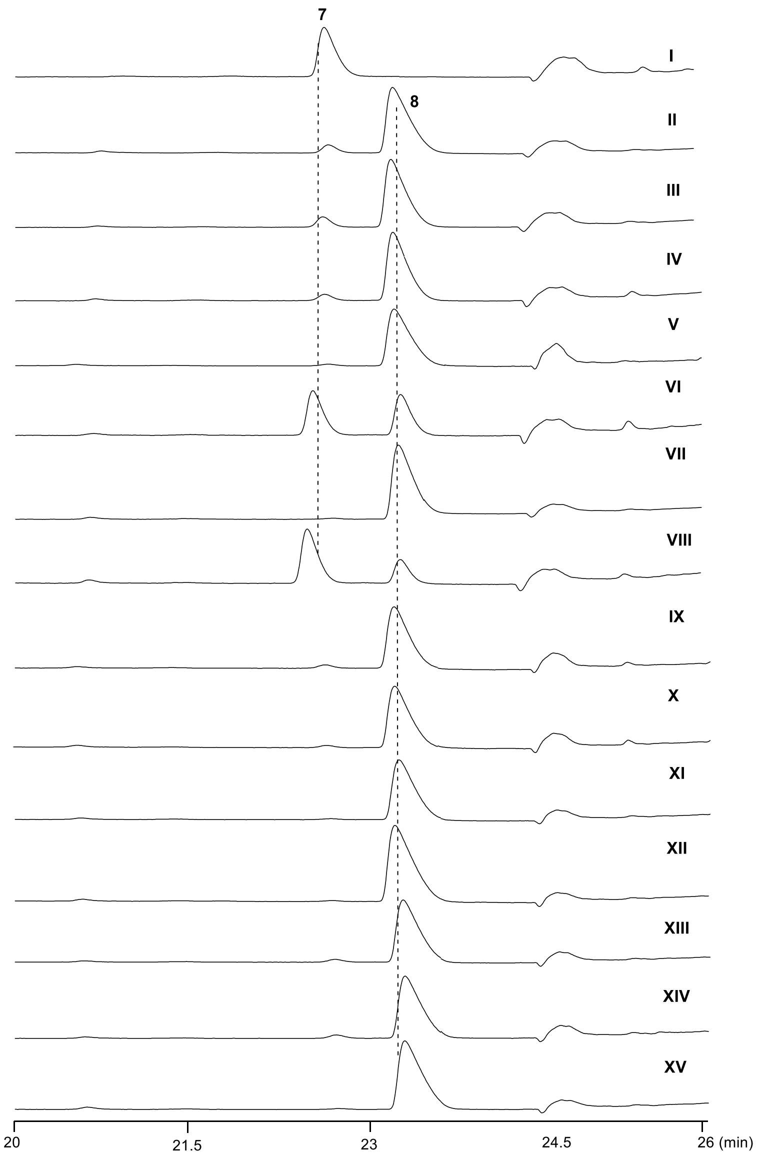

Supplementary Figure 25. Enzymatic assays of NapW and homologous proteins towards **7** were detected by HPLC analysis with UV absorption wavelength at 270 nm. (I) Substrate **7** + NADPH, (II) **7**+ NADPH + SDR-Pb, (III) **7** + NADPH + SDR-Cc, (IV) **7** + NADPH + SDR-Rm, (V) **7** + NADPH + SDR-Mb, (VI) **7** + NADPH+SDR-Li, (VII) **7** + NADPH + SDR-Cs, (VIII) **7** + NADPH + SDR-Ss, (IX) **7** + NADPH + NapW, (X) **7** + NADPH + homW, (XI) **7** + NADPH + SfmO1, (XII) **7** + NADPH + homO1a, (XIII) **7** + NADPH + homO1b, (XIV) **7** + NADPH + SlvW1, (XV) **7** + NADPH + SlvW2.

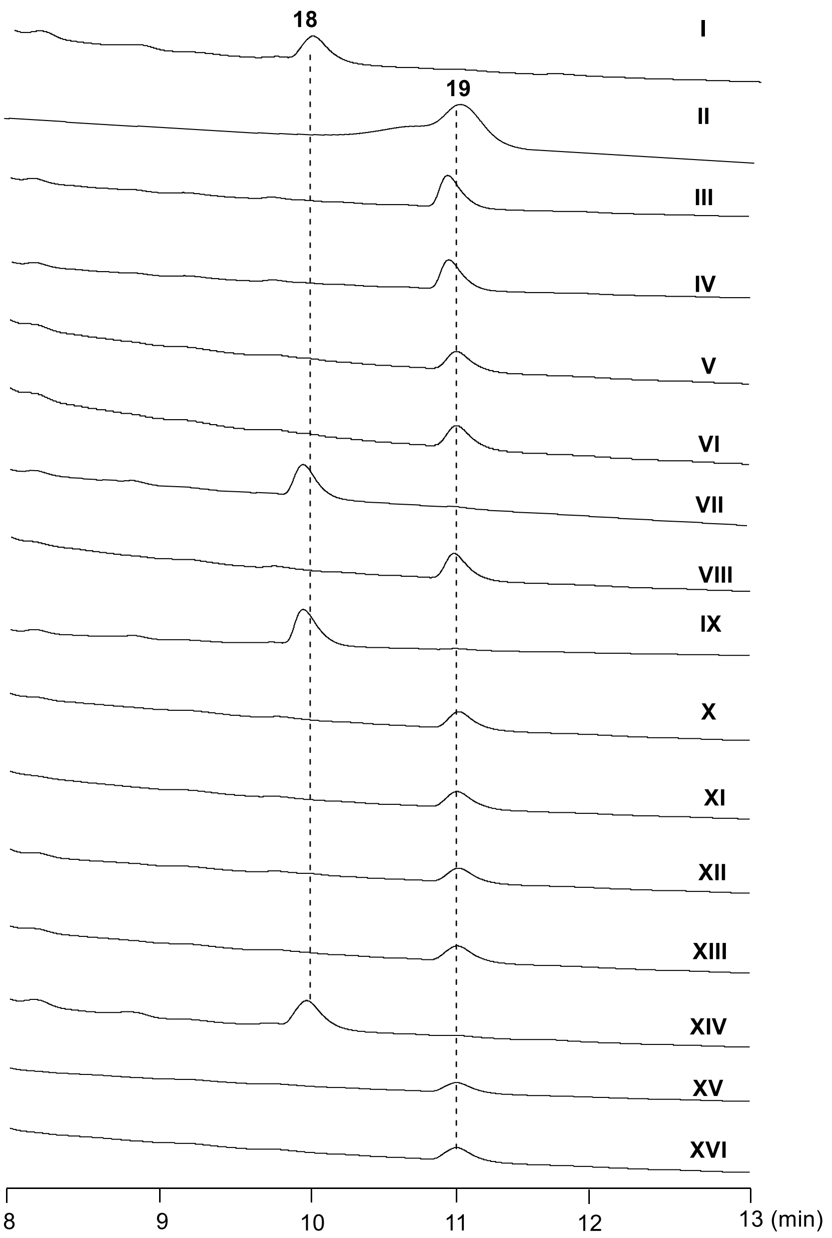

Supplementary Figure 26. Enzymatic assays of NapW and homologous proteins towards **18** were detected by HPLC analysis with UV absorption wavelength at 270 nm. (I) Substrate **18** + NADPH, (II) Standard **19**, (III) **18** + NADPH + SDR-Pb, (IV) **18** + NADPH + SDR-Cc, (V) **18** + NADPH + SDR-Rm, (VI) **18** + NADPH + SDR-Mb, (VII) **18** + NADPH+SDR-Li, (VIII) **18** + NADPH + SDR-Cs, (IX) **18** + NADPH + SDR-Ss, (X) **18** + NADPH + NapW, (XI) **18** + NADPH + homW, (XII) **18** + NADPH + SfmO1, (XIII) **18** + NADPH + homO1a, (XIV) **18** + NADPH + homO1b, (XV) **18** + NADPH + SlvW1, (XVI) **18** + NADPH + SlvW2.

Supplementary References

1. Whaley, H. A., Patterson, E. L., Dann, M., Shay, A. J. & Porter, J. N. Isolation and characterization of lemonomycin, a new antibiotic. *Antimicrob. Agents Chemother.* **14**, 83-86 (1964).
2. Huang, Y., Niu, B., Gao, Y., Fu, L. & Li, W. CD-HIT Suite: A web server for clustering and comparing biological sequences. *Bioinformatics* **26,** 680-682 (2010).
3. Gerlt, J. A. et al. Enzyme function initiative-enzyme similarity tool (EFI-EST): A web tool for generating protein sequence similarity networks. *Biochim. Biophys. Acta.* **1854**, 1019-1037 (2015).
4. Kieser, T., Bibb, M. J., Buttner, M. J., Chater, K. F. & Hopwood, D. A. Practical Streptomyces Genetics (John Innes Foundation, Norwich, UK) (2000).
5. Ye, Q. et al. Construction and co-expression of a polycistronic plasmid encoding carbonylreductase and glucose dehydrogenase for production of ethyl (*S*)-4-chloro-3-hydroxybutanoate. *Bioresour. Technol.* **101**, 6761-6767 (2010).
