# Supplementary material for: Reductive inactivation of the hemiaminal pharmacophore for resistance against tetrahydroisoquinoline antibiotics
Source: Nat Commun. 2021 Dec 6;12:7085. doi: 10.1038/s41467-021-27404-3 (PMC8648761; doi:10.1038/s41467-021-27404-3)
Supplement: Supplementary file 3 — Reporting Summary [file 41467_2021_27404_MOESM3_ESM.pdf]

nature portfolio

Corresponding author(s): Ting Shi, Jiahai Zhou, and Gong-Li Tang

Last updated by author(s): Oct 20, 2021

Reporting Summary

Nature Portfolio wishes to improve the reproducibility of the work that we publish. This form provides structure for consistency and transparency in reporting. For further information on Nature Portfolio policies, see our Editorial Policies and the Editorial Policy Checklist.

Statistics

For all statistical analyses, confirm that the following items are present in the figure legend, table legend, main text, or Methods section.

n/a	Confirmed	
☐	☒	The exact sample size (*n*) for each experimental group/condition, given as a discrete number and unit of measurement
☒	☐	A statement on whether measurements were taken from distinct samples or whether the same sample was measured repeatedly
☒	☐	The statistical test(s) used AND whether they are one- or two-sided<br>*Only common tests should be described solely by name; describe more complex techniques in the Methods section.*
☒	☐	A description of all covariates tested
☒	☐	A description of any assumptions or corrections, such as tests of normality and adjustment for multiple comparisons
☒	☐	A full description of the statistical parameters including central tendency (e.g. means) or other basic estimates (e.g. regression coefficient) AND variation (e.g. standard deviation) or associated estimates of uncertainty (e.g. confidence intervals)
☒	☐	For null hypothesis testing, the test statistic (e.g. *F*, *t*, *r*) with confidence intervals, effect sizes, degrees of freedom and *P* value noted<br>*Give P values as exact values whenever suitable.*
☒	☐	For Bayesian analysis, information on the choice of priors and Markov chain Monte Carlo settings
☒	☐	For hierarchical and complex designs, identification of the appropriate level for tests and full reporting of outcomes
☒	☐	Estimates of effect sizes (e.g. Cohen's *d*, Pearson's *r*), indicating how they were calculated

Our web collection on statistics for biologists contains articles on many of the points above.

Software and code

Policy information about availability of computer code

Data collection	All X-ray diffraction data were collected on beamlines BL17U1 and BL18U1 at the Shanghai Synchrotron Radiation Facility (SSRF).<br>MS data were collected and analyzed by Mass Spectrometer (Thermo Scientific LTQ XL).<br>UPLC/MSMS data were collected from Mass Spectrometer(Thermo vanquish orbitrap).<br>NMR data were collected from 600MHz (Bruker) and 500MHz (Agilent) NMR Spectrometer.
Data analysis	X-ray data were processed by HKL3000 and HKL2000.<br>Structure resolution was processed by Coot v0.8.9, CCP4 v7.0, Phenix v1.19.2-4158 and Pymol v2.4.1.<br>Chemical structures were drawn by ChemDraw v18.2.<br>MS data were processed by Xcalibur2.2.<br>NMR data were analyzed by Mestrenova 5.3.1 .

For manuscripts utilizing custom algorithms or software that are central to the research but not yet described in published literature, software must be made available to editors and reviewers. We strongly encourage code deposition in a community repository (e.g. GitHub). See the Nature Portfolio guidelines for submitting code & software for further information.

Data

Policy information about availability of data

All manuscripts must include a data availability statement. This statement should provide the following information, where applicable:

- Accession codes, unique identifiers, or web links for publicly available datasets
- A description of any restrictions on data availability
- For clinical datasets or third party data, please ensure that the statement adheres to our policy

Data supporting the findings of this work are available within the paper and its Supplementary Information file, which includes six tables (Table S1-S6) and twenty-six figures (Figure S1-S26). A reporting summary for this article is available as a Supplementary Information file. The genes homW, homO1a and homO1b generated in this study have been deposited in GenBank under the accession code MT230905 [https://www.ncbi. nlm.nih.gov/nuccore/MT230905.1], MT230906 [https://www.ncbi.nlm.nih.gov/nuccore/MT230906.1] and MT230907 [https://www.ncbi.nlm.nih.gov/nuccore/MT230907.1]. Structure data of NapW and NapW-NADP generated in this study have been deposited in the Protein Data Bank with the accession codes 7BTM [https://www.rcsb.org/structure/7BTM] and 7BSX [https://www.rcsb.org/structure/7BSX]. The raw data used for Supplementary Figs. 1, 2, 3, 6, 14, and 22 are provided in the Source Data file. The accession code, 2QQ5 [ttps://www.rcsb.org/structure/2QQ5] in Protein Data Bank was used as template. All other data that support the findings of this study are accessible in the manuscript and the Supplementary Information.

Field-specific reporting

Please select the one below that is the best fit for your research. If you are not sure, read the appropriate sections before making your selection.

☒ Life sciences ☐ Behavioural & social sciences ☐ Ecological, evolutionary & environmental sciences

For a reference copy of the document with all sections, see nature.com/documents/nr-reporting-summary-flat.pdf

Life sciences study design

All studies must disclose on these points even when the disclosure is negative.

Sample size	Quantitative experiments analyzed statistically for trend analysis are performed at triplicates, which is the standard sample size for biological experiment. Experiments not requiring statistical analysis were performed at least two independent experiments.
Data exclusions	No data were excluded.
Replication	Biological fermentation experiments were performed in triplicates. All enzyme assays and protein analysis experiments were verified with at least two independent enzyme preparations. All attempts at replication were successful.
Randomization	No randomization is needed for experimental setup.
Blinding	Chemical and biochemical experiments do not require blinded experiments, as the data is collected in an unbiased manner.

Reporting for specific materials, systems and methods

We require information from authors about some types of materials, experimental systems and methods used in many studies. Here, indicate whether each material, system or method listed is relevant to your study. If you are not sure if a list item applies to your research, read the appropriate section before selecting a response.

Materials & experimental systems

n/a	Involved in the study	
☒	☐	Antibodies
☒	☐	Eukaryotic cell lines
☒	☐	Palaeontology and archaeology
☒	☐	Animals and other organisms
☒	☐	Human research participants
☒	☐	Clinical data
☒	☐	Dual use research of concern

Methods

n/a	Involved in the study	
☒	☐	ChIP-seq
☒	☐	Flow cytometry
☒	☐	MRI-based neuroimaging
