# Supplementary material for: Reductive inactivation of the hemiaminal pharmacophore for resistance against tetrahydroisoquinoline antibiotics
Source: Nat Commun. 2021 Dec 6;12:7085. doi: 10.1038/s41467-021-27404-3 (PMC8648761; doi:10.1038/s41467-021-27404-3)

Reductive inactivation of the hemiaminal pharmacophore for resistance against tetrahydroisoquinoline antibiotics

Authors: Wan-Hong Wen[1], Yue Zhang[1], Ying-Ying Zhang[1], Qian Yu[2], Chu-Chu Jiang[2], Man-Cheng Tang[1], Jin-Yue Pu[1], Lian Wu[1], Yi-Lei Zhao[2], Ting Shi[2]*, Jiahai Zhou[3]*, and Gong-Li Tang[1,4]*

[1]State Key Laboratory of Bio-organic and Natural Products Chemistry, Center for Excellence in Molecular Synthesis, Shanghai Institute of Organic Chemistry, University of Chinese Academy of Sciences, Chinese Academy of Sciences, 345 Lingling Road, Shanghai 200032, China

[2]State Key Laboratory of Microbial Metabolism, Joint International Research Laboratory of Metabolic and Developmental Sciences, School of Life Sciences and Biotechnology, Shanghai Jiao Tong University, Shanghai 200240, China.

[3]CAS Key Laboratory of Quantitative Engineering Biology, Shenzhen Institute of Synthetic Biology, Shenzhen Institute of Advanced Technology, Chinese Academy of Sciences, Shenzhen 518055, China.

[4]School of Chemistry and Material Sciences, Hangzhou Institute for Advanced Study, University of Chinese Academy of Sciences, 1 Sub-lane Xiangshan, Hangzhou 310024, China

*Correspondence: Gong-Li Tang, Email: gltang@sioc.ac.cn

Jiahai Zhou, Email: jiahai@siat.ac.cn

Ting Shi, Email: tshi@sjtu.edu.cn

Source Data – Gel Images of DNA

Original File for Supplementary Figure 1

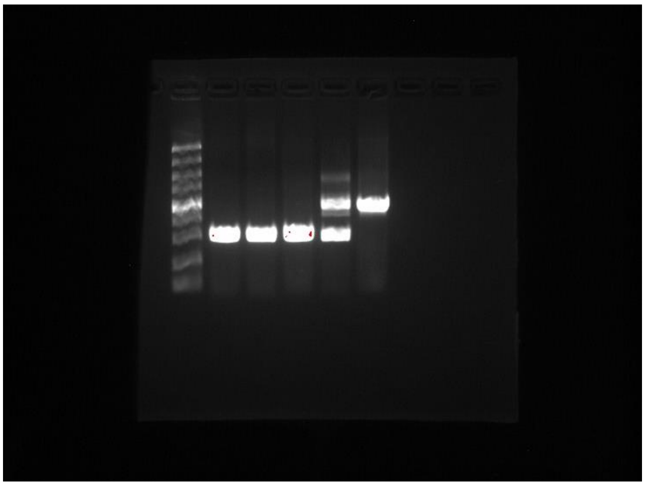

Original File for Supplementary Figure 2

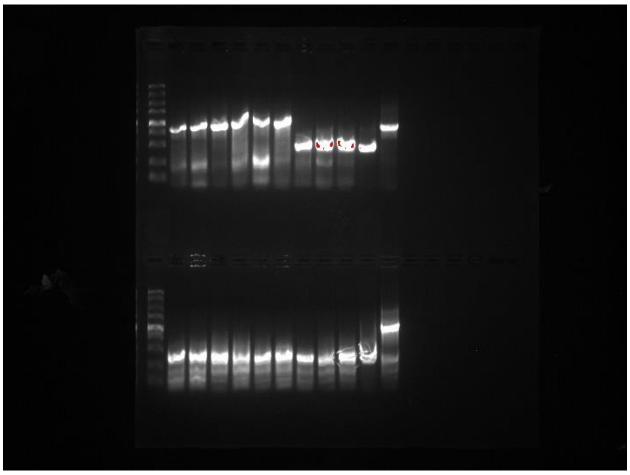

Source Data – Gel Images of protein

Original File for Supplementary Figure 3b

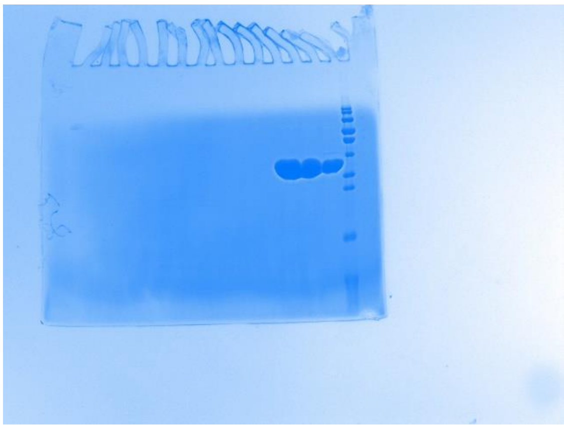

Original File for Supplementary Figure 3a, 14 and 22a

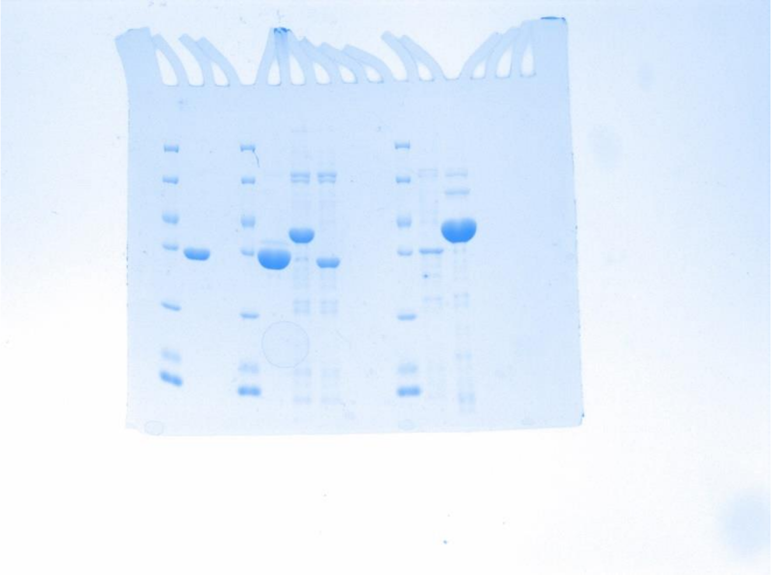

Original File for Supplementary Figure 6

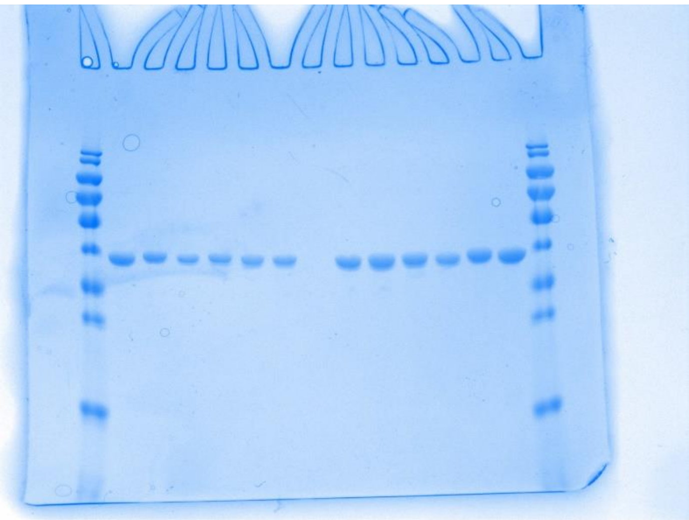

Original File for Supplementary Figure 22b

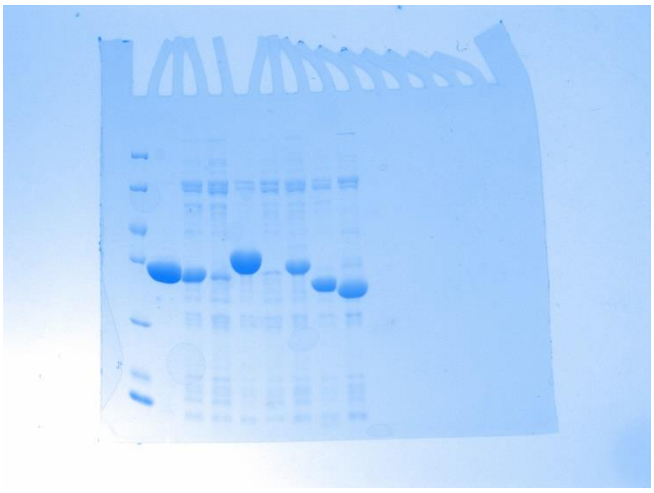

Supplement: Supplementary file 4 — Source Data [file 41467_2021_27404_MOESM4_ESM.pdf]
